# An interdisciplinary analysis of recreational birdwatching and wetland extent in the Murray-Darling Basin, Australia

**DOI:** 10.1016/j.isci.2026.114743

**Published:** 2026-01-19

**Authors:** James C.R. Smart, Jeremy Harte, Margaret Cook, Syezlin Hasan, J. Guy Castley, Alexandre Lima de F. Teixeira

**Affiliations:** 1 School of Environment and Science, Griffith University, Nathan, Brisbane, QLD 4111, Australia; 2 Australian Rivers Institute, Griffith University, Nathan, Brisbane, QLD 4111, Australia

**Keywords:** Ecology, Environmental management, Ornithology

## Abstract

To fulfill obligations under the Ramsar Convention and achieve the objectives of a National Biodiversity Strategy, the Australian Government has committed to improving the ecological characteristics of wetlands across the continental Murray-Darling Basin, Australia’s largest surface water resource. Allocating a portion of an increasingly climate-constrained water resource to environmental flows for this purpose necessitates careful evaluation of environmental and economic benefits. We combined a large citizen science dataset of birdwatching visitation over 72 consecutive months at ten Basin wetland birdwatching hotspots with spatiotemporal data on site-specific proxies of environmental and ecological condition. Using an interdisciplinary combination of regression analysis, citizen science data, an online birdwatchers’ survey and in-depth birdwatcher interviews, we found that improved ecological condition was associated with increased birdwatcher visitation. Our findings contribute to the policy debate by identifying increased birdwatching visitation and related expenditure as potential co-benefits of improving ecological condition at Basin wetland birdwatching hotspots.

## Highlights

• Birdwatcher visits and expenditure increase as condition of wetland sites improves
• Citizen science birdwatching data combined with a birdwatcher survey and interviews
• Contributes to policy debate around environmental flows by identifying co-benefits

## Graphical abstract

**Figure:**
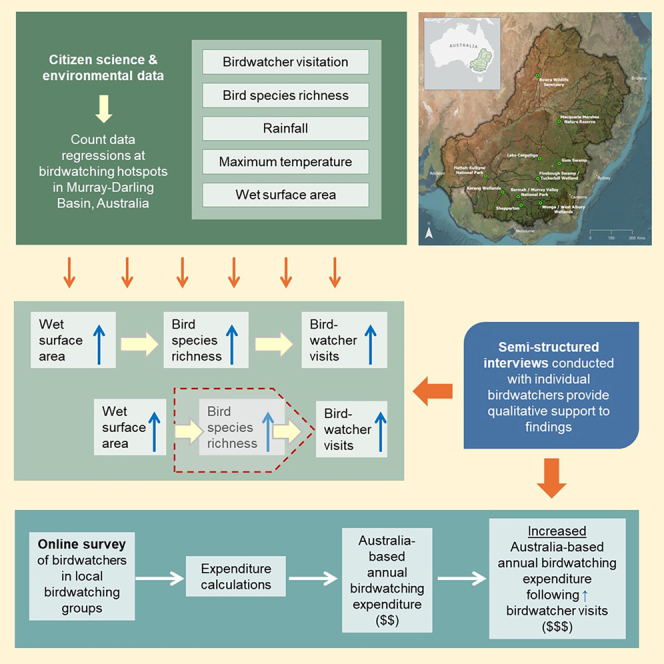

## Introduction

Australia has recently revised its National Biodiversity Strategy and Action Plan (NBSAP)^1^ to align more directly with the global biodiversity framework.^2^ The headline objectives of the NBSAP are to halt and reverse biodiversity loss by 2030 as the first step on a pathway to nature recovery by 2050. The Murray-Darling Basin (hereafter “the Basin”), an area spanning 1.06 million km^2^, is Australia’s largest surface water resource.^3^ The Basin contains more than 100 nationally important wetlands,^4^ of which 16 are listed under the Ramsar convention.^5^ To fulfill its international commitments under Ramsar, and meet the objectives of the NBSAP, the Australian Government has committed to releasing water flows from Basin storages (hereafter “environmental flows”) specifically to maintain the ecological character of targeted wetlands in the Basin.^1^^,^^6^

Under the Murray-Darling Basin Agreement,^7^ water is first reserved for critical human needs (drinking, sanitation, hygiene, and food preparation) and conveyance flows to maintain river connectivity. Portions of the remaining extractable volume are then allocated for irrigated agriculture and environmental flows. Irrigated agriculture and tourism are important sectors of the Basin’s economy.^8^ During FY19-20, the gross value of irrigated agricultural production in the Basin was AUD$6.39 billion.^9^ For the same financial year, the gross value added from tourism regions that intersect with the Basin was AUD$6.03 billion.^10^ Outdoor recreation in the form of day visits and overnight stays by domestic and international visitors is an important component of Basin tourism.^11^ Outdoor recreation is likely to be dependent, to some extent, on the condition of local ecological assets.^12^ Since wetlands are recognized as important birdwatching sites,^13^^,^^14^ birdwatching visitation would be expected to respond to improvements in wetland condition and bird occupancy.

The hydroclimatic future of the Basin, particularly the southern Basin where most irrigated agriculture and Ramsar wetlands are located, is predicted to become warmer and drier, with more severe droughts.^15^ This is likely to reduce water availability with consequent impacts on agricultural production, wetland condition, outdoor tourism, and the livelihoods and wellbeing of Basin communities.^15^^,^^16^ Equitable and efficient allocation of an increasingly climate-constrained water resource among competing uses, including environmental flows, necessitates careful evaluation of the environmental and economic benefits competing uses deliver.^17^ Recreational birdwatching, a cultural ecosystem service, would be expected to increase if bird occupancy or bird species richness increased following delivery of an environmental flow to a wetland birdwatching hotspot. However, this has not yet been demonstrated for wetland birdwatching sites in the Basin. Birdwatching visitation-related expenditures in local economies would be expected to increase thereafter as an economic co-benefit of improved ecological condition.

Following the Australian Bureau of Statistics (ABS), we regard *visitors* as “the central statistical entity in tourism statistics”,^18^ and define *domestic visitors* as Australian residents who travel outside their usual environment within Australia. The ABS define domestic visitors as either *overnight visitors*, who travel more than 40 kms from home and stay one or more nights at a location, or *same day visitors*, who travel over 50 kms in a round trip, outside of their usual environment.^18^ *International visitors* travel to a country other than the one in which they are usually resident.^18^

Traditionally, tourism visitation has been estimated via on-site, online or point-of-departure surveys,^19^ but these are time consuming and costly, particularly if data are to be collected at high temporal resolution across multiple sites. Alternatively, visitation data volunteered by members of the public via social media and citizen science platforms (termed volunteered geographic information (VGI))^20^ is being used increasingly to explore overall visitation rate,^21^ spatial and temporal variation in visitation,^22^ and for estimating the demand for, and valuation of, visitor experiences.^23^^,^^24^^,^^25^ Geo-located, time-stamped bird species sighting lists posted to the citizen science platform eBird^26^ have been used as VGI in economics-orientated visitation research.^23^^,^^24^^,^^27^

With the aim of quantifying whether birdwatching visitation co-benefits are associated with improved ecological condition at wetland sites, we applied three complementary research approaches: regression analysis, an online survey, and face-to-face interviews. Drawing on a large multi-year citizen science dataset on birdwatching visitation at ten wetland birdwatching hotspots across the Basin, we investigated the following research questions.

• Can regression analysis establish an association between the ecological condition of wetland birdwatching hotspots (proxied by water surface area) in the Murray-Darling Basin and birdwatcher visitation rates to those sites (proxied via eBird-derived VGI)?
• If so, when paired with data on per-visit-day Basin birdwatching-related expenditures from a birdwatchers’ survey, can indicative estimates of the additional birdwatching-related economic activity associated with improving the ecological condition of birdwatching hotspot sites in the Basin be estimated?
• When expressed in their own words through face-to-face interviews, do birdwatchers’ motivations for visiting birdwatching destinations in the Basin support findings from our quantitative analysis?

Our regression results indicated that increased water surface area at a wetland birdwatching hotspot is associated with increased monthly birdwatcher visitation both *directly* (i.e., visitation increases in the month immediately following an increase in water surface area) and *indirectly* (i.e., visitation increases two months after an increase in water surface area, following an increase in bird species richness in the intervening month). Research results enabled us to predict the percentage increases in birdwatcher visitation associated with a given increase in wet surface area at hotspot sites, together with consequent increases in birdwatching-related expenditures. Birdwatcher interviews provided qualitative support, in birdwatchers’ own words, for the associations identified in our regression analyses.

## Results

### Drivers of birdwatching visitation

**Figure 1:**
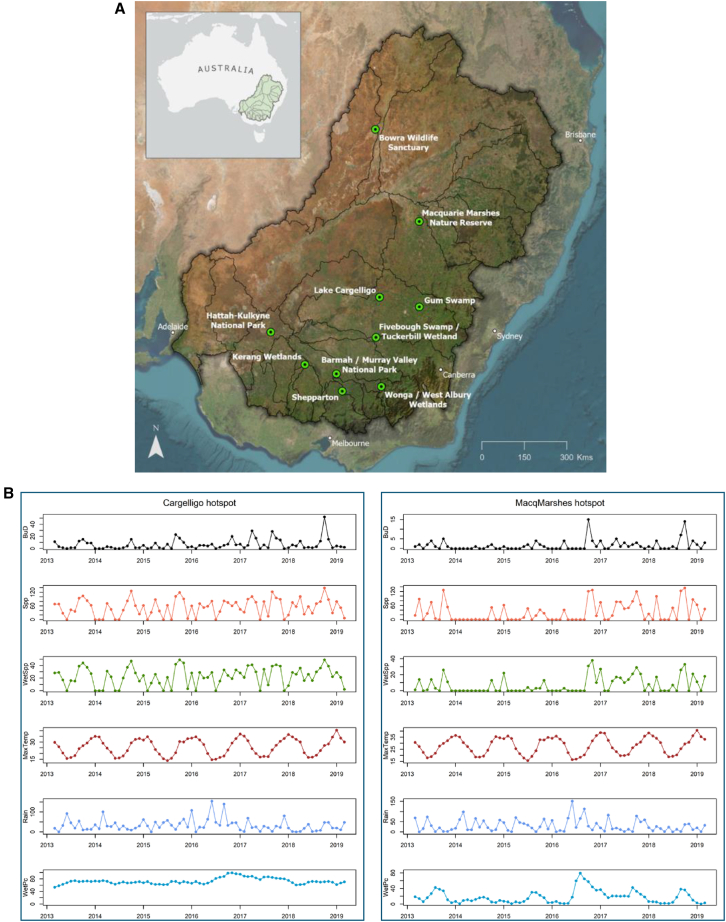
Hotspot locations, monthly variation in birdwatcher visitation, bird species richness, and environmental and ecological characteristics (A) Birdwatching hotspots across the Murray-Darling Basin, identified from species list postings on eBird and our birdwatcher survey. (B) For two indicative hotspots: monthly variation in birdwatcher visitation (proxied by eBird BuDs) (black), eBird-reported species richness for all birds (orange) and water and water-edge habitat-related bird species (green), average daily maximum temperature (dark red), total rainfall (light blue), and percentage of maximum observed wet surface area for on-site waterbodies (turquoise).

Count data regressions^28^^,^^29^ were used to identify potential drivers of birdwatching visitation at ten wetland birdwatching hotspots in the Basin (Figure 1A). A proxy for birdwatcher visitation at each wetland hotspot was generated using VGI from bird sighting checklists posted on the eBird citizen science platform.^26^ eBird checklists are tagged with an anonymous username unique to each birdwatcher (“eBirder”) who posts checklists on eBird. Posted checklists report bird sightings by species, location, date and time. Separate bird species list postings at a hotspot were grouped into checklist posts per individual eBirder per visit day. These are termed “Birder User Days” (BuDs). Monthly BuD counts were used as a proxy for monthly birdwatcher visitation at each birdwatching hotspot. BuD counts at each hotspot were compiled for 72 consecutive months from 1 April 2013 to 31 March 2019.

**Figure 2:**
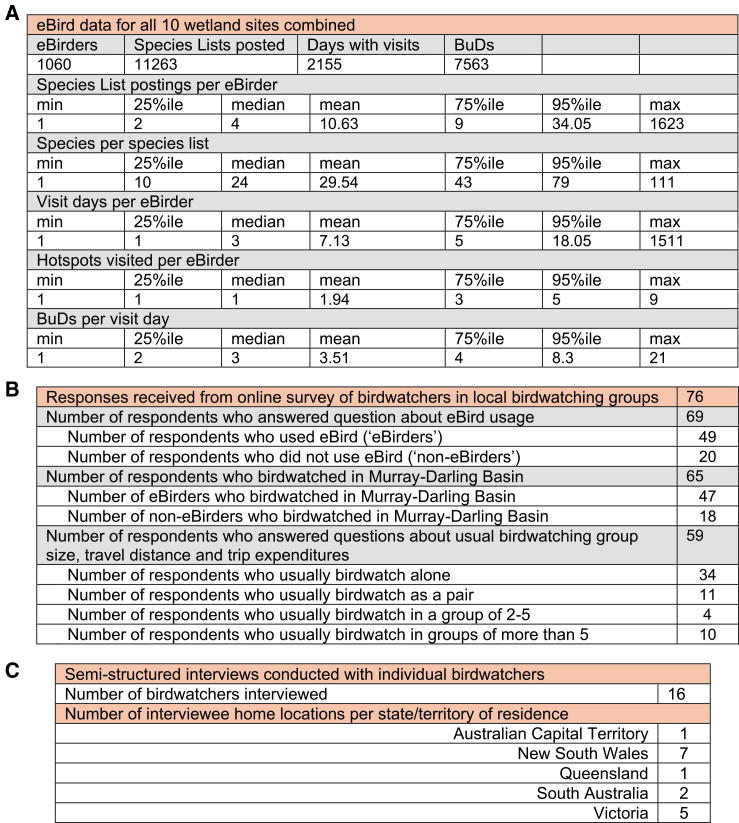
Descriptive statistics data from our three data sources (A) Data from eBird bird species list postings used for count data regressions on potential drivers of birdwatcher visitation and eBird-derived bird species richness at monthly resolution. Descriptive statistics reported for data derived from all eBird species list postings from our 10 wetland sites between 1 April 2013 and 31 March 2019 (2191 days) (see Table S1 for descriptive statistics for eBird-derived data for individual hotspots). (B) Responses from our online survey of Birdwatchers in local birdwatching groups in New South Wales, Victoria, South Australia, and the Australian Capital Territory. Survey responses were used to estimate the ratio of eBird users to non-eBird users, the number and duration of day trips and overnight trips to birdwatching sites in the Murray-Darling Basin annually, usual traveling group size, and typical expenditures incurred on those trips. (C) Semi-structured interviews with individual birdwatchers. Quotations from these interviews provide qualitative support, in birdwatchers’ own words, for the associations identified in our regression analyses.

A total of 11263 bird species sighting checklists were posted to eBird by 1060 individual eBirders who visited our ten wetland hotspot sites over the 72-month timescale of our analysis (Figure 2A). Across all ten sites the mean number of species list postings per eBirder was 10.63, and the mean number of bird species per posted species list was 29.54. The average number of wetland hotspot visit days per eBirder was 7.13, and the mean number of hotspots visited by eBirders was 1.94. Descriptive statistics for eBird posts from each individual hotspot site are shown in Table S1.

**Figure 3:**
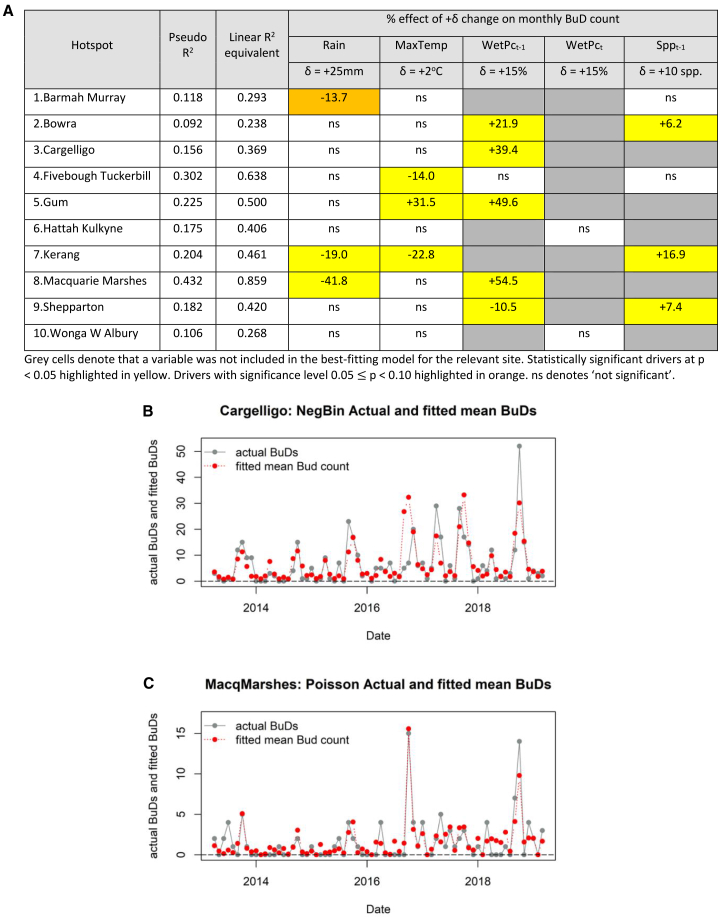
Regression results and indicative fitted vs. actual plots for count data models of monthly BuD counts at individual hotspots (A) Regression results for time-varying drivers in best-fitting individual hotspot models of monthly BuD counts. (B) Fitted mean vs. actual monthly BuD counts from the best-fitting negative binomial model for Lake Cargelligo hotspot. (C) Fitted mean vs. actual monthly BuD counts from best-fitting Poisson model for the Macquarie Marshes hotspot. (See Figures S4 and S6 for the remaining eight hotspots).

In our first set of regressions we used monthly BuD counts as the dependent variable, with monthly variations in environmental and ecological parameters, month-of-the-year and an overall month-index as potential drivers (Figures 1B and S1–S3, and Table S2 and STAR Methods). When analyzed separately as individual hotspots, a statistically significant *direct* association between water surface area in the *preceding* month and birdwatching visitation during the *current* month was identified in the best-fitting regression models at five hotspot sites (Figure 3A and STAR Methods). For four sites, higher water surface area during the *preceding* month was significantly associated with higher BuD visitation during the *current* month. In the Shepparton model, the polarity of action was reversed. Most hotspot-specific BuD models produced a good fit to observed BuD data, evidenced by fitted vs. actual plots, pseudo-R^2^ fit statistics and their linear R^2^ equivalents (Figures 3B and 3C, 3A, and S4–S6, Table S3). Figure 3A shows that water surface area from the *preceding* month was found to be statistically significant in five of the six hotspot-specific best-fitting models in which it featured, whereas the *current* month’s water surface area featured in only two hotspot-specific best-fitting models, but not significantly so.

The influences of hotspot-specific month-of-the-year effects in combination with hotspot-specific time trends are evident in the fitted vs. actual plots (Figures 3B, 3C, and S4–S6). Month-of-the-year terms for six hotspots showed similar patterns, with visitation typically peaking in October (the spring peak in bird breeding) and remaining high over the summer holiday period before dropping substantially during the winter (Figures S11A, S11C, S11D, and S11F–S11H). Month-of-the-year effects for Shepparton and Wonga West Albury Wetlands share some of these features, but visitation at these sites dropped off less over the winter (Figures S11I–S11J). Month-of-the-year effects at Bowra reflect the annual closure of the wildlife sanctuary at this site from mid-October to April. Multi-year time trends differed considerably between hotspots, with some sites showing substantial increase in monthly BuD counts across the model time span (Figure S12). This could be due to underlying increases in birdwatcher visitation, or increasing popularity of eBird, or both. Hotspot-specific autocorrelation plots (Figures S7–S10) show that, in combination, month-of-the-year and the time trend account adequately for temporal correlation in BuD counts.

Inclusion of current monthly total rainfall and average daily maximum temperature, and reported overall bird species richness from the *preceding* month, improved the predictive performance of some hotspot-specific BuD models (Figure 3A). The signs of the rainfall and maximum temperature terms (when significant) generally matched *a priori* expectation; birdwatchers being less likely to visit in hotter or wetter conditions. So too did the sign of reported species richness from the *preceding* month. Interviews with birdwatchers revealed that some consult recent species listings on eBird or other birdwatching apps when choosing destinations for their next birdwatching trip.

When modeled across the combined ten-hotspot panel (Figure S13), higher bird species richness in the *preceding* month and lower *current* month rainfall were both statistically significantly associated with an increase in *current* month BuD count. However, neither water surface area from the preceding month, nor water surface area from the current month, were significant drivers of current month BuD count in the ten-hotspot panel model. The month-of-the-year term in the panel model retained several of the features present in the individual hotspot models (Figures S15A and S11). The time trend in the BuD panel model falls within the range of time trends from the individual hotspot models (Figure S15B).

### Drivers of reported bird species richness

Count data regressions for hotspot sites individually and across the combined ten-site panel were used to identify significant drivers of monthly variation in eBird-reported bird species richness (for all birds) and eBird-reported species richness of water and water-edge habitat-related birds at hotspot sites.^30^ Monthly site-specific counts of bird species richness were constructed by summing the number of unique bird species appearing on all eBird species checklists posted from a specific wetland in a particular month. (See STAR Methods for details, and Table S5 for full listings of unique species reported at each hotspot site). Percentage wet surface area during the *current* month *or* percentage of wet surface area during the *preceding* month, monthly total rainfall, monthly average maximum daily temperature, month-of-the-year, and a time trend were included as potential drivers.

A ‘long list’ of bird species utilizing freshwater or freshwater-edge habitats was constructed using species included in the following categorizations from Garnett et al.: Feeding habitats—Inland waters, rivers and streams, deep open waters, shallow open waters, reeds and tall wet grassland, low marshland and wet grassland; breeding habitats—inland wetland.^30^ Site-specific monthly eBird species lists were checked for the presence of water and water-edge habitat-related species from this ‘long list’ to produce site-specific monthly counts of species richness for water and water-edge habitat-related species. (See Table S6 for the ‘long list’ of species potentially utilizing freshwater or freshwater-edge habitats).

The action of significant terms in the best-fitting hotspot-specific bird species richness models largely matched *a priori* expectation (Figures S16A and S16B). More rain and higher temperatures generally act to reduce reported species richness, presumably by deterring birdwatcher visits (although temperature effects at Gum Swamp appear anomalous). Increased wet percentage area in the *preceding* month is significantly positively associated with increased reported species richness in the *current* month in three of the best-fitting site-specific models for overall bird species richness, and in five of the best-fitting site-specific models for richness of water and water-edge habitat-related species (Figures S16A and S16B). In contrast, although the best-fitting model of overall bird species richness for Shepparton does include wet percentage area in the *current* month, this term is not significant. The best-fitting model for water and water-edge habitat-related bird species richness for Wonga West Albury does include wet percentage area in the *current* month; however, at this site, this driver acts to reduce species richness (Figure S16B). Also, as would be expected, percentage wet area in the preceding month is particularly influential in models of water-related bird species richness. The fit of the species richness models is much lower than that of the BuD models (Figures S16A, S16B, and 3A). This is not surprising as eBird-reported species richness will include substantial sources of unobserved variation. For example, the time that a birdwatcher spends at a site and their expertize in species identification will likely affect the number of species in the species list that birdwatcher posts on eBird. However, these sources of variation are not available in our data.

Results from the ten-site panel regression models of bird species richness show that wet surface area in the *preceding* month and wet surface area in the *current* month are both statistically significantly associated with current month species richness (all birds) and current month species richness of water-related species (Figures S13 and S14). However, overall model fit, and the statistical significance of the water surface area term, is consistently higher when wet surface area in the *preceding* month is included as a driver.

### Increased water surface area is associated with higher species richness and higher birdwatcher visitation

**Figure 4:**
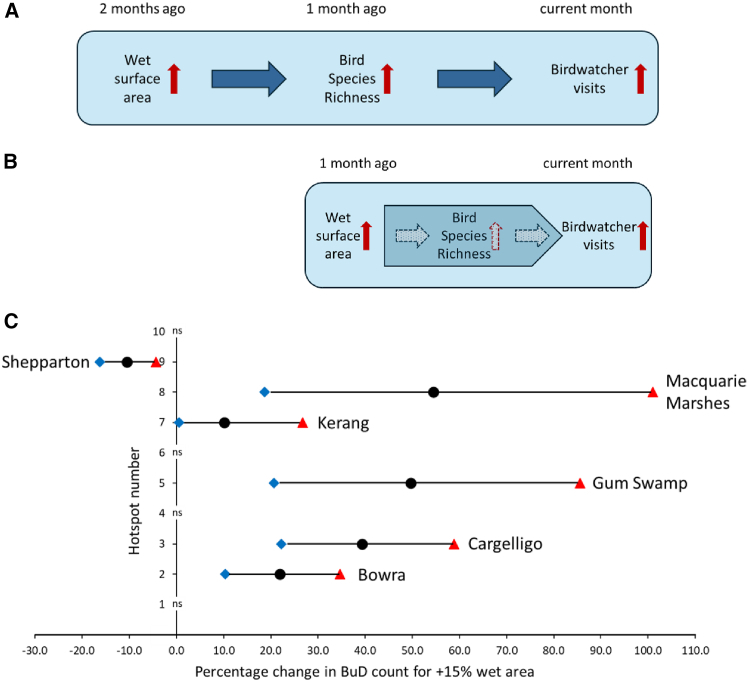
Association chains and expected changes in BuD count following a 15% increase in wet surface area at individual hotspot sites (A) Two-step association: an increase in wet surface area at a hotspot two months ago is associated with increased eBird-reported bird species richness one month ago, which is then associated with increased birdwatcher visitation in the current month. (B) Single-step association: an increase in wet surface area at a hotspot one month ago is associated with increased birdwatcher visitation in the current month. (C) Expected percentage changes in BuD count at individual hotspot sites following a 15% increase in wet surface area during an earlier month. Black circles denote median predicted changes, blue diamonds 5^th^ percentiles, and red triangles 95^th^ percentiles. “ns” denotes that wet percentage area was not a statistically significant term in the best-fitting count data model for the site. At Kerang Wetlands the relationship between wet area and BuD count is mediated through an increase in bird species richness in the intervening month (Figures 3A and S16A).

Results from the panel models for eBird-reported species richness and monthly BuD counts support a statistically significant *indirect* association between increased water surface area and increased birdwatcher visitation, mediated through an increase in bird species richness. An increase in wet surface area two months ago is statistically significantly associated with increased overall bird species richness one month ago (Figure S13B), which is then statistically significantly associated with an increase in BuD visitation during the current month (Figure S13A). This two-step linkage was also present in the individual hotspot model for Kerang (Figure S16A for wet surface area to species richness; Figure 3A for species richness to BuD count). These findings are consistent with a two-step association between increased water surface area and increased birdwatcher visitation (Figure 4A).

In addition, BuD models for five individual hotspots (Bowra, Lake Cargelligo, Gum Swamp, Macquarie Marshes, and Shepparton) identified a statistically significant *direct* association between increased wet surface area one month ago and BuD visitation during the current month (Figure 3A). These findings are consistent with the single-step association in Figure 4B. The two-step association in Figure 4A could collapse to the single-step association in Figure 4B if a hotspot’s ecological response time (between expanding area of water and water-edge habitats and increased bird species richness), followed by birdwatchers’ response time to app alerts of increased bird species richness, are sufficiently rapid to compress both steps of Figure 4A into the same month. These responses should be invoked irrespective of whether the increase in wet surface area arises because of rainfall events or targeted environmental flows.

Estimated regression parameters can be used to calculate the increases in BuD count that would be expected to follow from a given percentage increase in wet surface area at a hotspot site.^29^ For the five hotspots where wet surface area during the *preceding* month was a significant *direct* driver of BuD count during the following month (through association chain 4B), a 15% increase in wet surface area in the *preceding* month would be expected to produce changes of between −10.5% and +54.5% in the following month’s BuD count (Figure 4C), assuming all other drivers remain unchanged. For Kerang Wetlands, where the expected increase in BuD count is mediated via an increase in bird species richness in the intervening month (association chain 4A), a 15% increase in wet surface area would be expected to change BuD count two months thereafter by +10.2% (Figure 4C).

Using parameter estimates from the panel models (Figures S13 and S14), following the association in Figure 4A, and assuming all other drivers remain unchanged, a 15% increase in wet surface area two months prior would be expected to change hotspot BuD count during the current month by +6.7%.

### Birdwatching expenditure

We ran an online survey on birdwatching activities and expenditures with members of local birdwatching groups in New South Wales, Victoria, South Australia, and the Australian Capital Territory (ACT). Survey responses were used to estimate, for Australia-based birdwatchers in those states and the ACT, the ratio of eBird users to non-eBird users, the number and duration of day trips and overnight trips taken annually to birdwatching sites in the Murray-Darling Basin, usual traveling group size, and typical expenditures incurred on those trips. Methods and supplemental text section S2 explain how these results were obtained from online survey responses. Figure 2B reports the number of survey responses received.

A total of 76 surveys were returned. Of these 59 provided sufficient information for complete analysis. Almost equal proportions of responses were received from birdwatchers resident within and outside the Basin (49% and 51%, respectively, Figure S17). Survey responses provided information on the number of day and overnight birdwatching trips group members made to the Basin annually, whether they usually traveled alone or as part of a group (of a particular size), and the typical expenditures incurred (Figure S18). Among survey respondents who went birdwatching in the Basin, 72.3% used eBird. To the best of our knowledge, no other survey has quantified the proportion of eBird users in a sample of birdwatchers who go birdwatching in the Murray-Darling Basin. Hence, we use the 72.3% eBird usage from our survey to estimate the total number of birdwatching visits by non-eBird users at hotspot sites.

**Figure 5:**
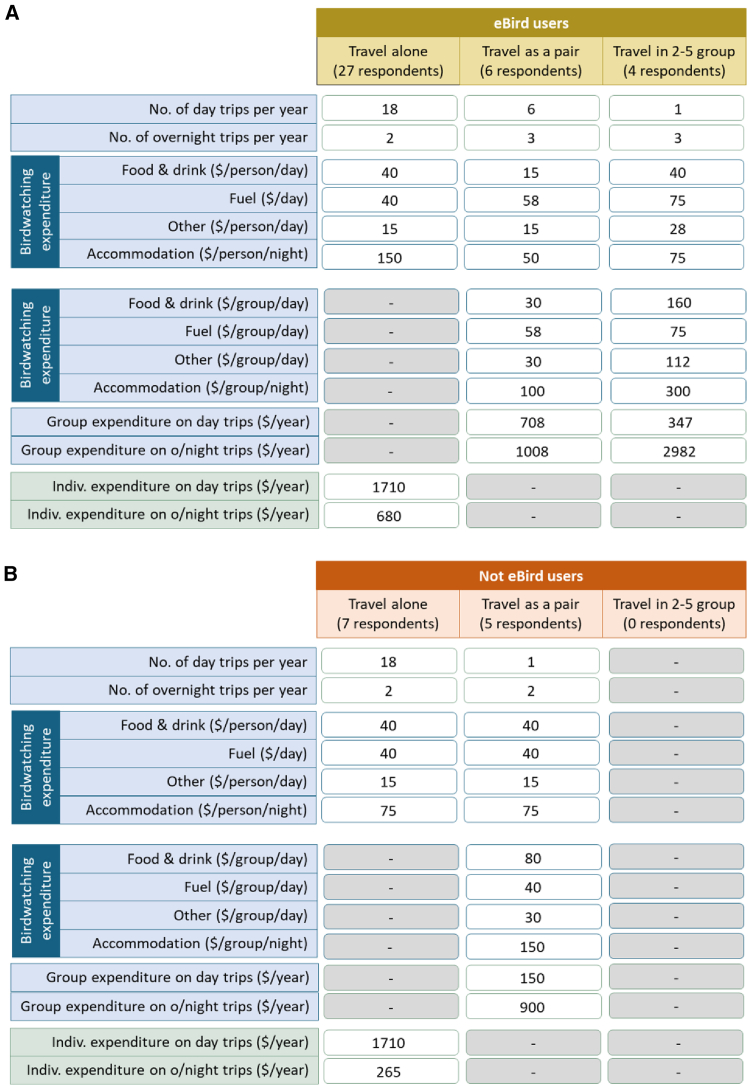
Birdwatching activities and birdwatching-related expenditures by our survey sample of Australia-based birdwatchers active in the Basin (A) Birdwatching activities and expenditures by Basin eBirders. (B) Birdwatching activities and expenditures by Basin non-eBirders. Expenditure totals (green rows) produced using medians of reported survey responses (blue rows). See STAR Methods and supplemental text section S2 for further details of these calculations.

**Figure 6:**
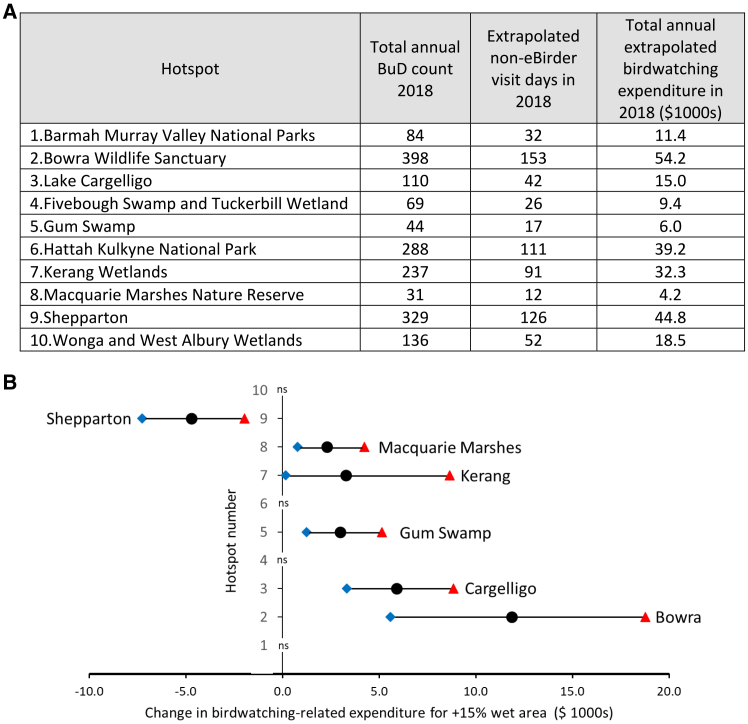
Estimated annual birdwatching-related expenditures by Australian-based birdwatchers at hotspot sites and predicted expenditure increases associated with a 15% increase in wet surface area (A) Estimated hotspot-specific total annual birdwatching-related expenditures by Australia-based birdwatchers at 10 wetland birdwatching hotspots during 2018. (B) Predicted expenditure increases by Australia-based birdwatchers associated with a 15% increase in wet surface area at hotspot sites for which wet surface area is statistically significantly associated with birdwatching visitation, directly or indirectly. Black circles denote median predicted changes, blue diamonds 5^th^ percentiles, and red triangles 95^th^ percentiles. “ns” denotes that wet percentage area was not a statistically significant term in the best-fitting count data model for the site. At Kerang, the predicted increase in birdwatching expenditure includes the effect of increased wet area two months ago on species richness one month ago, and the subsequent effect of increased species richness on increased BuD count in the current month.

Based on median responses to survey questions on expenditures, numbers of day and overnight trips, and traveling group size (Figures 5 and S20), per-visit-day activity-weighted expenditures were AUD$ 98.11 for Australia-based eBirders and AUD$ 99.27 for Australia-based non-eBirders who birdwatch in the Basin (STAR Methods). We did not include expenditure data from the 10 survey respondents who said they normally traveled in groups larger than 5 in our expenditure calculations because we were not convinced that expenditure data from a single respondent were necessarily representative of expenditures by all group members in these larger groups. These per-visit-day expenditures were applied to hotspot total annual BuD counts from 2018 (the most recent complete year in our data), and extrapolated numbers of non-eBirder visit days, to estimate the annual expenditure injected into local economies from birdwatching visitation by Australia-based birdwatchers’ visits during 2018 (Figure 6A, STAR Methods). Across our ten hotspot sites, annual birdwatching-related expenditures by Australia-based birdwatchers during 2018 were predicted to be between $4,200 and $54,200.

Median survey responses were used to estimate per-visit-day activity-weighted expenditures because a modest proportion of survey respondents reported Basin birdwatching visit frequencies of “more than two trips a week”, which we took to be 144 trips per year (3 trips per week for 48 weeks over one year, excluding Christmas and Easter holiday periods. (See supplemental text section S2 for details of how annual numbers of visits were estimated from categorical survey data). Taking the annual number of Basin birdwatching trips made by eBirders who travel alone as an example, five respondents reported taking “more than two trips a week”. This produced a highly skewed distribution of annual number of visits with a mean (37 trips per year) to median (18 trips per year) ratio of 2.06. (For comparison, the 75th percentile in these data were 39 trips per year). Median values were therefore used as the basis for annual expenditure calculations to produce conservative expenditure estimates that should be relatively robust against inclusion or exclusion of a small number of respondents with very high levels of birdwatching engagement.

The same approach was used together with predictions of expected percentage increases in BuD count from the individual hotspot regressions (Figure 4C), to calculate the increases in Australia-based birdwatchers’ 2018 expenditures that would be expected to follow from a 15% increase in wet surface area at relevant hotspot sites (Figure 6C, Table S9) (assuming all other drivers of birdwatching visitation remain unchanged, and that expenditures increase in proportion to the change in overall birdwatcher visitation). Across the six hotspot sites where wet surface area was significantly associated with birdwatcher visitation, either directly or indirectly, annual Australia-based birdwatching-related expenditures during 2018 would be expected to change by amounts of between −$4,700 and +$11,900 following a 15% increase in water surface area, with expenditure increases expected at five of these six sites.

### In birdwatchers’ own words

Semi-structured interviews were conducted with 16 individual Basin birdwatchers (Figure 2C, STAR Methods). Thematic analysis of interview transcripts and direct quotations from interviews provide qualitative support, in birdwatchers’ own words, for the associations identified in our regression analyses.

Thematic analysis of interview transcripts clearly established that riparian habitats and wetlands across the Basin are important birdwatching sites. Surface water was widely recognized as a bird attractant and interviewees believed that increases in water coverage sparked rapid increases in the number and variety of birds. A birdwatcher in Mildura, Victoria said: “*many [types] of birds rely on the river system and if we get rainfall or floods like we had two years ago, that brings in more water sources for bird*s. *We have those massive breeding events. It’s amazing to see*”*.* According to a birdwatcher from Horsham, Victoria “*water is important*”. They explained that a few years ago Dock Lake in Horsham was dry, but “*now it has water for the first time, I think, in about 12 years. It’s had it now for two or three years. The bird life has exploded there*”. Our Horsham interviewee told us when water arrives “*thousands and thousands of birds come in. It’s incredible to see how it does attract the birds and support birds there, including breeding*”*.*

Some interviewees considered environmental flows to be essential for birds, birdwatchers and the health of riparian zones. Interviewees typically identified sites that offered ideal habitat (often sites with water), numerous birds, good facilities and accommodation options as their favorite birdwatching locations. A birdwatcher in Condobolin, New South Wales told us: “*Environmental water has been fantastic and it’s making a big difference to the bird population*”*.* Gaynor Swamp Wildlife Reserve is one of the favorite birdwatching places of a birdwatcher from Mooroopna, Victoria as it has 10 wetlands and receives environmental flows. Our Mooroopna interviewee describes environmental water as “*the best thing ever*”. But they told us it needs to be managed to reflect the seasonality of wetlands and swamps. For example, our Mooroopna interviewee does not want Gemmill Swamp permanently watered “*because most of our wetlands need to dry out*”. A birdwatcher from Wirrate, Victoria maintained that environmental water “*is essential*”. Our Condobolin interviewee said the environmental water allocated to the Lachlan River in Condobolin “*has been fantastic and it’s making a big difference to the bird population. Environmental water is vitally important for bird numbers along the river*”. Our Condobolin interviewee told us that floods are crucial too, and recounted that the 2022 flood “*brought huge bird breeding events*”. Along the Lachlan River our Condobolin interviewee recalled they had 20,000 pelicans breeding as it is the “*biggest brooding area in New South Wales*” for pelicans. “*It’s an inland river that requires a flood to make it happen*”. They also had 60,000 ibis brooding at Booligal on the Lachlan to the north of Condobolin. The concentration of water reflected the natural water flows before dam construction. As our Condobolin interviewee noted “*that’s why there is a big swamp on the bottom of the Lachlan, which is a world-renowned site for water birds because that’s where all the water used to go. So we’re going to try to get a little bit of water into there on occasions and they need to keep that going*”.

For a birdwatcher from Sydney, New South Wales, water can “*often be the first focal point*” for choosing where to go birdwatching but “*then it is a question of, well, that’s fantastic, that really looks great, but what else is there within reasonable distance that could add to the interest*”?. Conversely, the absence of water can ruin a birdwatching trip. For example, a birdwatcher from Cobram, Victoria went to The Coorong where the River Murray meets the sea in March 2024 and it was “*really dry and there was barely anything. I think I saw pelicans that were really about all. It was very, very quiet bird-wise*”.

Through social media apps such as eBird and via word of mouth (including through bird club networks), news would spread quickly and visiting birdwatchers would soon follow the birds. Our Horsham interviewee told us: “*We knew it wasn’t keen migration season, we just wanted to go and explore. I used eBird and Birdata before we started booking anything just to sort of see what sorts of things have been reported up there at that time of year*”. A Mildura bird watcher told us “*I will use eBird to look at the hot spots or where species have been seen and go out to those areas*”.

While birdwatching can be a solitary activity (which was an attraction for some), many enjoyed the social elements: club membership, social media, group activities and the connectivity of eBird or Birdata (a birdwatching app from Birdlife Australia). Our Condobolin interviewee said: “*it’s a good network to be in because everyone shares. It’s not like fishermen who won’t tell you their best spot. If someone sees a good bird they’ll tell everybody. No one cares if someone goes and gets a good photo of it. Or sees it. It’s really sharing in that department*”. Our interviewees in Condobolin, Mildura, and Cobram, all keen photographers, commented that after posting bird photos on social media people contact them to see where the photo was taken, which creates a community and also attracts visitors to the place. Our Condobolin interviewee admitted that getting people to visit Condobolin and spend money was one of the reasons he posted.

Connectivity on social media is a big part of birdwatching culture. Unlike in the past, social media means “*people are connected all over the world, not just in their local tiny town*”, as said by a birdwatcher in Canberra in the Australian Capital Territory, so people may not feel the need to join a club. Another birdwatcher from Sydney enjoys the online social engagement: “*I belong to a few birding Facebook groups and there’s a lot of people that are passionate about photographing birds and they take fantastic photos. It’s just another way of remembering your experiences and what you’ve seen*”. A member of the Mildura bird club shared that the club’s Facebook page has “*about 500 or 600 people on it. We have people join all the time from all over the country and say ‘I’m coming to Mildura. What places do you guys suggest that I go birding”? and we give people places to go*”.

Interviewees’ birding expenditures ranged from fuel and grocery purchases on small-scale camping trips to organized groups of birdwatchers catching planes, hiring cars, staying in hotels and eating in local restaurants over trips lasting several days. Many interviewees thought that birdwatching in Australia was increasing in popularity, and some felt that local communities failed to capitalize fully on the economic potential of this market, particularly in small rural towns. A birdwatcher in Illawarra, New South Wales, went with 26 people to Darwin for a week. “*Nearly all of them flew, only a couple of us drove. Everybody spent several thousand dollars*”.

Our Canberra interviewee said the Canberra birdwatching club has about 370 members, equal numbers of men and women, with “*fewer people under 40 than over 60. Having been a birder all my life, I would say younger people are birding just as much, possibly even more, than when I was young*”. But things have changed with the “*gig economy for work*”, poorer wages, precarious employment, and social media. “*They’re birding and communicating just as much but just not joining clubs in a traditional way*”. While older people might join the clubs, our interviewee from Horsham knew that 30-year-olds are bird watching. “*They might not necessarily be in the clubs because of other time constraints, but I am hearing that it's quite popular among young people*”. There was a consensus that they birdwatched in other ways. Our Cobram interviewee noted that it is “*certainly encouraging to see some more younger people out there*”, and they said it is especially popular with young males.

Another key theme was the mental health benefits of bird watching. As our interviewee from Condobolin shared, “*it’s great for your well-being, it’s certainly a great way to spend your time. You get away from everything, forget about things, get out and have a look around at nature*”. Our Mildura interviewee agreed that bird watching “*just takes you away from the normal life it’s very healing. It gets you a bit out of your own head*”. Our Horsham interviewee commented that “*when you’re doing it, you’re not thinking about anything else. It really is a break from everything else that you’re doing because you are focused on it. You’re listening intently for all the clues, you’re watching intently. All your senses are really engaged. From a mental health perspective, I think, for me personally, it’s really beneficial*”. Some interviewees mentioned that bird watching was a great hobby for neurodivergent people and it is great for relieving stress. Many commented that during the Covid19 lockdowns birdwatching grew in popularity and offered solace to many people who were largely indoors. Our Illawarra interviewee summarized what they think is important about birdwatching. “*It has three elements to it. It’s mentally challenging, it’s socially interactive because you’re talking to other birdwatchers regularly, and then you’re out in nature*”.

In combination, findings from our three research approaches evidence that increased surface water area at wetland birdwatching hotspots across the Basin is generally associated with increased birdwatcher visitation, which in turn increases birdwatching-related expenditure in local economies.

## Discussion

The eBird citizen science VGI dataset in our analysis recorded 7563 birdwatching visit days in total, identified from 11263 geo-located, time-stamped species list posts by 1060 eBirders over 72 consecutive months across ten wetland sites (Figure 2A) at no cost to the research project. It would have been prohibitively expensive to collect an equivalent dataset by traditional on-site surveys. Combining this large panel visitation dataset with spatiotemporal data on site-specific proxies of environmental and ecological condition, allowed us to identify that changes in condition are associated with changes in birdwatcher visitation at wetland birdwatching hotspots across the Basin. Our findings contribute to the policy debate by identifying increased birdwatching visitation and related expenditure as potential co-benefits of improving ecological condition at Basin wetland birdwatching hotspots.

While the representativeness of data from citizen science and social media platforms can be affected by factors such as the age profile of platform users, the popularity of sites being visited and temporal variation in the popularity of the platforms themselves,^31^ the authors are not aware of any other data source that could provide six consecutive years of monthly resolution, site-specific data on birdwatcher visitation and bird species richness for birdwatching hotspots in the Basin. A recent study produced “home country” estimates from global postings of eBird species lists from individual eBirders during a 2-year window prior to the date on which they posted a bird species list from Ramsar-listed Gunbower National Park and Koondrook-Perricoota State Forest wetlands on the Murray River.^32^ These sites are approximately 35 km and 125 km by road, respectively, from the Kerang Lakes and Barmah Forest Ramsar sites in this study. Results from the Gunbower, Koondrook-Perricoota eBird-based analysis (Table S7) show that Australia-based eBirders accounted for at least 83% of eBirder visitation, and that the 82 eBirders who visited the site between 1 April 2013 and 31 March 2019 posted a total of 18,849 species lists globally during the 2-year windows prior to their visit; an average of 115 species list posts per eBirder annually. This level of posting activity suggests that many of the eBirders who visit the Basin’s wetlands are likely to be avid birdwatchers, so eBird-derived monthly bird species richness data should be relatively reliable.

Our online survey of 76 Australia-based birdwatchers in local birdwatching groups yielded 59 fully usable responses which, together with in-depth telephone interviews with 16 individual birdwatchers, provided confirmatory evidence to support findings from our quantitative analyses. Similar interdisciplinary approaches could be applied to advance application of large-scale spatiotemporal citizen science VGI datasets in other policy settings. Citizen science data on birdwatching activities are readily available,^26^ but the possibilities of applying VGI data to estimate economic returns from investments to improve the condition of natural assets that are relevant for other forms of outdoor recreation should also be explored.

Birdwatchers are known to have diverse motivations^33^^,^^34^; however, bird species richness is well established as a desirable characteristic of birdwatching sites,^35^ and this was confirmed through our interviews with individual birdwatchers. The eBirder to non-eBirder ratio used to extrapolate eBird-derived BuD counts to a total number of birdwatching visit days is highly influential over our predicted expenditure increases. So too is our use of birdwatching expenditures derived from a survey of Australia-based birdwatchers who are members of local birdwatching groups. Among our survey respondents, activity-weighted per-visit-day average expenditures are modest at $98/day and $99/day, respectively, for eBirders and non-eBirders active in the Basin. When per visit day expenditures for eBirders and non-eBirders combined are calculated separately (i.e., when activity weighting is removed), expenditures for day-trip days ($88.39 ± $3.53 (95% CI) and overnight-trip-days ($207.78 ± $14.44 (95% CI)) are similar to the day-trip (2024 AUD$109) and overnight-trip-day (2024 AUD$223) expenditures for domestic birdwatchers reported in Tourism Research Australia’s (TRA’s) Domestic National Visitor Survey for FY2018-19, as quoted by Steven^36^ (See supplemental text section S2 and Figure S23). This comparison suggests that, while not identical, expenditure results from our small visitor survey of birdwatchers active in the Basin are comparable with average all-of-Australia expenditure results from TRA’s FY2018-19 Domestic National Visitor Survey (as quoted in Steven^36^) which reported 516,000 birdwatching day trips and 331,000 birdwatching overnight trips taken by domestic visitors. (TRA’s Domestic National Visitor Survey included birdwatching as a category of outdoor recreation from 2019 onwards.^36^).

Among our survey respondents, day visits comprised 92% of visitation (Figure 5). Most of our predicted expenditure increases following an improvement in ecological condition at hotspot sites thus come from day visits. TRA’s domestic visitor survey for FY2018-19 reported that day trips comprised 61% of domestic birdwatching-related tourism, with overnight trips comprising the remaining 39%.^36^ This is almost a 5-fold increase in the proportion of overnight trips compared with results from our survey of members of local birdwatching groups who go birdwatching in the Basin. With average daily expenditure on overnight trips being more than twice as high as average day visit expenditure in our survey (and in TRA’s FY2018-19 data), a higher proportion of overnight trips would substantially increase domestic birdwatchers’ contributions to local economies.

International birdwatchers spend considerably more per trip-day than domestic birdwatchers.^37^ In addition to evoking a quasi-local visitation response from Australia-based birdwatchers, environmental watering to maintain or improve the ecological characteristics of important wetlands, particularly the Basin’s Important Bird Areas,^38^ could potentially consolidate Basin wetland sites in the travel itineraries of these higher-expenditure visitors.^39^ If so, this would be expected to substantially increase the longer-term economic co-benefits of environmental watering at Basin wetland sites. TRA data report that birdwatching-related travel expenditure from domestic overnight visitors totaled $636 million during FY2023-24, whereas birdwatching-related expenditure from international visitors totaled $2.6 billion for the same financial year.^40^ Quantification of the economic co-benefits of environmental watering of the Basin’s wetlands from this source remains an opportunity for further research.

Our research focused on increased birdwatching visitation expenditure as a co-benefit of improving the ecological condition of Basin wetlands. If improving the ecological condition of wetlands acts to increase the overall level of birdwatching activity, co-benefits will also arise through improvements in physical and mental health.^36^ Several of our interviewees told us that birdwatching was important for improving their physical and mental health, and for maintaining social connectedness and well-being.

To fulfill its obligations under Ramsar and the NBSAP, the Australian Government has committed to maintaining and improving the ecological characteristics of wetland sites across the Basin.^1^^,^^6^ Environmental flows play an important role in delivering this outcome.^6^ By applying an interdisciplinary research approach to a large citizen science spatiotemporal VGI dataset this research has established that economic co-benefits likely follow from environmental flows via associated increases in recreational birdwatching, a cultural ecosystem service, at Basin birdwatching hotpots. Further research is required to quantify the full extent of these co-benefits, particularly those generated by longer-distance visitors; however, the findings reported here are an important first step in estimating these outcomes.

### Limitations of the study

Our count data regression is based on monthly data from 1 April 2013 to 31 March 2019. Literature suggests that visitation patterns and numbers for outdoor recreation, including birdwatching, have changed considerably post-COVID.^41^ A post-COVID repeat of our analysis might therefore be worthwhile. Our study applied the proportion of eBird usage from our own 59-respondent survey when estimating total birdwatcher expenditures at hotspot sites in the Murray-Darling Basin. Unfortunately, we cannot check the representativeness of eBird usage among our survey respondents relative to the wider population of birdwatchers in the Basin due to the lack of comparable information from other data sources. TRA’s Domestic National Visitor Survey on Australian residents’ participation in all forms of visitation (e.g., holidays, visiting family and friends, business travel, attending sporting events etc.) included birdwatching as a separate category of outdoor recreation from 2019,^36^ however, a question on respondents’ usage of birdwatching apps is not included in the TRA survey, nor are socio-demographic or socio-economic characteristics provided for domestic visitors who indicate they have engaged in birdwatching. Similarly, when estimating birdwatching-related expenditures at our hotspot study sites we based these on per-day expenditures and the proportion of day trips to overnight trips from our birdwatchers’ survey rather than equivalent results from TRA’s Domestic National Visitor Survey. We did this because our results are derived from birdwatchers known to be active in the Basin, and because they provided more conservative expenditure estimates.

## Resource availability

### Lead contact

Requests for further information and resources should be directed to and will be fulfilled by the lead contact James C. R. Smart (j.smart@griffith.edu.au).

### Materials availability

Birdwatcher visitation and bird species counts data were derived from ebird.org data. The Cornell Lab of Ornithology (https://www.birds.cornell.edu/home/) approved use of eBird data for this research under specified Terms of Use (https://www.birds.cornell.edu/home/ebird-data-access-terms-of-use/). Derived data on numbers of eBirder visit days and the number of bird species reported (for all bird species and, separately, for water-related bird species) at monthly resolution for each of our 10 wetland hotspot sites over the six-year study period are provided as a supplementary Excel file. The standard eBird Terms of Use also apply to these derived data.

Survey and interview data were provided voluntarily by members of local birdwatching groups. The collection, use, storage and final destruction of these data were covered by Human Research Ethics Approval 2024/212, granted by Griffith University Human Research Ethics Committee. Human Research Ethics Approval 2024/212 required that primary data obtained “will be retained for 5 years by Griffith University and then destroyed.” This condition was included in the Project Information Sheet provided to survey/interview participants during the recruitment and consent process. To comply with the terms under which participants agreed to be surveyed/interviewed, and in accordance with Griffith University Human Research Ethics Approval 2024/212, primary survey and interview data cannot be made available.

### Data and code availability

• Data on daily rainfall, daily maximum temperature and wet surface area of waterbodies reported in this paper will be shared by the lead contact upon request.
• This paper does not report original code.
• Any additional information required to reanalyze the data reported in this paper is available from the lead contact upon request.

## Acknowledgments

This work was undertaken as a part of the Murray-Darling Basin Water and Environment Research Program (MD-WERP) Social, Economic and Cultural Outcomes Theme. The MD-WERP is an Australian Government initiative to strengthen scientific knowledge of the Murray-Darling Basin that is managed through a partnership between the Murray-Darling Basin Authority (MDBA), the Commonwealth Environmental Water Office (CEWO), and the 10.13039/501100024290Department of Climate Change, Energy, the Environment and Water (DCCEEW). The authors pay respect to the Traditional Owners and their Nations of the Murray-Darling Basin. We acknowledge their deep cultural, social, environmental, spiritual, and economic connection to their lands and waters.

The authors thank 10.13039/100010337Birdlife Australia (www.birdlife.org.au) for arranging for organizers of Birdlife Australia local groups to publicize this research and distribute the link to the online data collection survey to local birdwatching group members. The authors particularly thank Erin Farley (Birdlife Australia, Campaigns and Participation Program Leader at the time of this study) for helpful comments and suggestions regarding survey design and distribution. Thanks to all the people who completed the questionnaire on Survey Monkey and for the generosity of the birdwatchers who were interviewed and enriched the study.

eBird citizen science data were obtained for research use, with authorization, from the Cornell Lab of Ornithology, 159 Sapsucker Woods Road, Ithaca, NY, 14850, USA; ebird@cornell.edu.^26^ The authors particularly thank Jenna Curtis (eBird Project Leader) for helpful comments and suggestions.

## Author contributions

Conceptualization, J.C.R.S. and S.H.; methodology, J.C.R.S., J.H., and S.H.; investigation, J.C.R.S., J.H., M.C., S.H., J.G.C., and A.L.F.T.; writing—original draft, J.C.R.S., M.C., and S.H.; writing review & editing, J.C.R.S., S.H., M.C., J.G.C., J.H., and A.L.F.T.; funding acquisition, J.C.R.S.; resources, J.C.R.S., J.H., and M.C.; supervision, J.C.R.S.

## Declaration of interests

The authors declare no competing interests.

## STAR★Methods

### Key resources table

**Table:** 

REAGENT or RESOURCE	SOURCE	IDENTIFIER
**Deposited data**		

Survey and interview data provided by members of local birdwatching groups	This paper	Retained at Griffith University under Human Research Ethics Approval 2024/212.

**Software and algorithms**		

STATA/SE® v15.1.	Stata Corp^42^	http://www.stata.com
Survey Monkey	N/A	www.surveymonkey.com
Esri’s ArcGIS Pro 3.4. Released 7 November 2024.	N/A	https://pro.arcgis.com/en/pro-app/latest/get-started/download-arcgis-pro.htm
R version 4.4.1 (2024-06-14 ucrt) -- “Race for Your Life”	Copyright (C) 2024 The R Foundation for Statistical Computing Platform: x86_64-w64-mingw32/x64	https://www.r-project.org
RStudio 2023.09.1+494 “Desert Sunflower” Release (cd7011dce393115d3a7c3db799dda4b1c7e88711, 2023-10-16) for windows.	© 2009-2023 Posit Software, PBC	https://posit.co or https://posit.co/products/open-source/rstudio/?sid=1

### Method details

#### Hotspot selection

Hotspots of birdwatching visitation were identified by a combination of spatially plotting monthly BuD counts across the Basin, spatial hotspot analysis in Esri’s ArcGIS, and a ranked listing of most frequently-visited Basin birdwatching sites from the online survey with members local birdwatching groups. This produced the ten hotspot locations shown in Figure 1A.

#### Data

##### Birdwatcher visitation rate

The eBird site operators (Cornell Lab of Ornithology)^26^ encourage use of eBird data for scientific research and granted permission for eBird data to be used in this research. Bird species checklists posted to eBird report bird sightings by species, location, date and time. Following standard practice in analysing VGI,^31^ separate bird species list postings at a hotspot were grouped into checklist posts per individual eBirder per visit day and termed ‘Birder User Days’ (BuDs). Monthly BuD counts were used as a proxy for monthly birdwatcher visitation at each birdwatching hotspot. BuD counts at each hotspot were compiled for 72 consecutive months from 1 April 2013 to 31 March 2019. Amalgamating individual posts into ‘visitor days’ to use as a proxy for visitation rate has been found to be reasonably reliable,^31^ although reliability can be affected by factors such as the overall popularity of the location, the age profile of visitors to that location and temporal variation in the popularity of the citizen science platform.

##### Bird species richness

eBird-reported bird species richness (total number of different bird species) is included as a potential driver of monthly BuD count, and as the dependent variable in separate regression analyses. Bird species richness for a month is reported as a simple count of the total number of unique bird species reported in all eBird species listings posted from the hotspot site that month. Bird species richness is reported separately for all bird species and for bird species that use freshwater and freshwater-edge habitats for feeding or breeding, where a ‘long list’ of Australian bird species that use freshwater or freshwater-edge habitats is compiled from Garnett et al. (Table S6).^30^

##### Rainfall and maximum temperature

Daily rainfall (mm) and daily maximum temperature data (^o^C) data were obtained at 5.6km x 5.6km grid resolution from the Australian Bureau of Meteorology. For hotspots spanning more than one grid cell, (i) daily rainfall averaged across all intersecting grid cells was summed across the month to produce the monthly total rainfall (mm) for the site, and (ii) daily maximum temperature was averaged across all of the hotspot’s grid cells and then averaged across the month to produce the average daily maximum temperature for the hotspot in that month.

##### Wet surface area of water bodies

All hotspot sites contain several waterbodies listed in Digital Earth Australia’s Waterbodies dataset (Table S4).The combined wet surface area of all waterbodies at a hotspot in each month, reported as a percentage of the summed maximum observed wet surface area for all Digital Earth Australia-listed waterbodies at the hotspot site over the period 1 April 2013 to 31 March 2019, was used as a proxy for wetland condition.^43^^,^^44^ Cloud cover can obscure remote sensing of wet surface area.^45^ Wet surface area data were linearly interpolated across cloud-induced breaks in the dataset for months when less than 50% of the maximum total observed wet surface area was visible.

##### Regression modelling method

Monthly BuD counts and monthly eBird-reported bird species richness can only be zero or positive whole numbers. Count data regression models were therefore used to identify their potential drivers.^28^^,^^29^^,^^46^ The Poisson count regression model for monthly BuD count at an individual hotspot is:

$${BuD}_{t}∼Poisson\left(\mu_{t}\right)$$

$$E\left({BuD}_{t}\right)=\mu_{t}\;\text{and}\;var\left({BuD}_{t}\right)=\mu_{t}$$

$$\ln\left(\mu_{t\;}\right)=\beta_{1}+\beta_{2}{Rain}_{t}+\beta_{3}{MTemp}_{t}+\beta_{4}{WetPxPc}_{t}\;\text{or}\;\beta_{5}{WetPxPc}_{t-1}+\beta_{6}Sp{pCt}_{t-1}$$

$$+\beta_{7f}{Month\_factor}_{t}\;{+\beta }_{8}{Month\_index}_{t}$$

Driving factors enter as independent variables which can influence (the natural log of) the expected mean (*μ*_*t*_) BuD count for each monthly timestep (*BuD*_*t*_): total monthly rainfall (*Rain*_*t*_), average maximum daily temperature through the month (*MTemp*_*t*_), percentage of summed maximum observed wet area (*WetPxPc*_*t*_), and the eBird-reported bird species richness (all species) from the *preceding* month (*SppCt*_*t*-1_). Percentage wet area enters as *either* percentage wet area for the *current* month (*WetPxPc*_*t*_) *or* for the *preceding* month (*WetPxPc*_*t*-1_). Month-of-the-year is introduced as a categorical factor (*Month*_*factor*_*t*_). Month index (counting 1 to 72) (*Month*_*index*_*t*_) is also included. These variables provide the ability to represent monthly cycles and a temporal trend.

Equivalent count data models were used to identify significant drivers of monthly eBird-reported bird species richness (*SppCt*_*t*_). Separate models were constructed to identify significant drivers of monthly species richness (all birds) and monthly species richness of water-related birds. Negative binomial models^28^^,^^29^ for BuD counts and bird species richness were also estimated for all individual sites to account for data that contain a wider range of monthly count values.

Problematic multicollinearity was screened for using variance inflation factors (VIFs).^47^ Variables with VIFs higher than 3 were not entered into the same regression equation.^46^ Multicollinearity between percentage wet area during the *current* month and the *preceding* month prevented these two drivers being included in the same model. Hotspot-specific count data models for BuD count were therefore estimated with the following combinations of percentage wet area and the preceding month’s reported species richness as potential drivers:

1. Percentage wet area for the current month (alone)
2. Percentage wet area for the preceding month (alone)
3. Species richness (all bird species) for the preceding month (alone)
4. Percentage wet area for the current month and Species richness for the preceding month
5. Percentage wet area for the preceding month and Species richness for the preceding month

Similarly, the following combinations of drivers were evaluated for modelling hotspot-specific bird species richness (for all species and for water habitat-related species, separately):

1. Percentage wet area for the current month (alone)
2. Percentage wet area for the preceding month (alone)

Monthly total rainfall, maximum monthly temperature, month-of-the-year, and month-index terms were included in all BuD count and species richness models.

Results from the best-fitting of models 1 to 5 for BuD counts and the better-fitting of models 1 and 2 for species richness, in their most appropriate functional form (Poisson or negative binomial), are reported separately for each hotspot. The most appropriate functional form was determined via a Likelihood Ratio test of the overdispersion parameter from the negative binomial regression.^29^

Poisson-form fixed effect panel data models were used to identify significant drivers of variation in BuD counts and bird species richness (all species and water-related species) across the ten-hotspot combined panel.^28^^,^^48^^,^^49^ Monthly total rainfall, maximum monthly temperature, month-of-the-year, and month-index terms were included as potential drivers in all panel models. Water surface area from the preceding month *or* from the current month were introduced separately as potential drivers.

Hotspot-specific and panel data models were implemented in STATA/SE v15.1.^42^ Hotspot-specific models were estimated using STATA’s glm routine with Newey-West Heteroskedasticity and AutoCorrelation robust (HAC) standard errors to ensure robust statistical inference.^50^ Panel data models were estimated using STATA’s xtpoisson fixed effects routine using robust bootstrapped standard errors with bootstrap replications clustered by hotspot. Statistical significance of individual drivers at p < 0.05 and 0.05 ≤ p < 0.10 levels are flagged in yellow and orange, respectively, in tables of regression results.

##### Birdwatcher survey

The online survey was implemented using Survey Monkey^51^ after obtaining ethics approval (Griffith University Reference Number: 2024/212). The ethics approval also covered the birdwatcher interviews. A link to the survey was distributed by Birdlife Australia Local Groups and birdwatching clubs in New South Wales, Victoria, the Australian Capital Territory and South Australia via birdwatching group emails and newsletters. Birdwatching group members volunteered by clicking on the survey link and confirming their consent to participate. Survey participants were thus members of local birdwatching groups, over 18 years of age, resident in Australia, who had signed up to receive birdwatching group newsletters via email and were active birdwatchers. The survey questions were constructed to allow direct comparison with survey data collected for a previous Birdlife Australia bird tourism report.^52^

Survey questions elicited information on general birdwatching activities, levels of engagement with the eBird citizen science app, birdwatching activities in the Basin including typical expenditures on accommodation, fuel, food and drink, park entry fees and tour guides, whether birdwatching is typically undertaken alone or in a group, and the distance travelled for a typical trip. Survey data were collected from 1 August 2024 to 8 September 2024. An overview of the survey questions is provided in Figure S18.

Results from the online birdwatcher survey allow us to calculate the proportions of eBirder respondents who usually travel alone (73%), as a pair (16%), or as a group of 2-5 (11%), along with the proportions of day trips relative to overnight trips taken by each travelling group size (90% of trips taken by lone travellers are day trips, 67% of trips taken by travelling pairs are day trips, 25% of trips taken by groups of 2-5 are day trips).This information allows the overall proportions of Murray-Darling Basin birdwatching activity undertaken by each category combination (lone travellers on day trips, lone travellers on overnight trips, pair travellers on day trips, pair travellers on overnight trips etc.) of eBirders within the survey sample to be calculated. Overall eBirder activity proportions are reported in Figure S21. Information reported in Figure 5 allows the overall proportions of Murray-Darling Basin birdwatching activity undertaken by each category combination of non-eBirders to be calculated (Figure S21).

Annual activity proportions of day and overnight trips for each traveller category (Figure S21), are combined with the total annual day trip and overnight trip expenditures by traveller category from Figure 5 to calculate the annual activity-weighted birdwatching expenditure associated with a representative eBirder from our sample who goes birdwatching in the Murray-Darling Basin. These calculations are as follows:

Annual trip activity-weighted expenditure associated with a representative MDB eBirder visiting MDB ($/year) =

• Lone traveller day trip expenditure = $1710/year x 0.657 = $1123/year +
• Lone traveller overnight trip expenditure = $680/year x 0.073 = $50/year +
• Pair traveller day trip expenditure = $708/year x 0.108 = $76/year +
• Pair traveller overnight trip expenditure = $1008/year x 0.054 = $54/year +
• 2-5 group traveller day trip expenditure = $347/year x 0.027 = $9/year +
• 2-5 group traveller overnight trip expenditure = $2982/year x 0.081 = $242/year

Annual trip activity-weighted expenditure associated with a representative MDB eBirder from our sample = **$1554/year**

Similarly, combining non-eBirders’ annual activity proportions from Figure S21 with non-eBirders’ total annual day trip and overnight trip expenditures by traveller category from Figure 5, allows us to calculate the annual activity-weighted birdwatching expenditure associated with a representative non-eBirder from our sample who goes birdwatching in the Murray-Darling Basin, as follows:

Annual trip activity-weighted expenditure associated with a representative MDB non-eBirder visiting MDB ($/year) =

• Lone traveller day trip expenditure = $1710/year x 0.525 = $898/year +
• Lone traveller overnight trip expenditure = $265/year x 0.058 = $15/year +
• Pair traveller day trip expenditure = $150/year x 0.139 = $21/year +
• Pair traveller overnight trip expenditure = $900/year x 0.278 = $250/year

Annual trip activity-weighted expenditure associated with a representative MDB eBirder from our sample = **$1184/year**

When combined with the per person, per day expenditures incurred by different travelling group categories from Figure 5, the annual activity proportions for day and overnight birdwatching trips for the different group sizes (Figure S21) allow per-visit-day activity-weighted expenditures to be calculated across travelling group categories for eBirders and non-eBirders who go birdwatching in the Murray-Darling Basin. These calculations produce the following results:

Per-visit-day activity-weighted expenditure associated with a representative MDB eBirder in our sample ($/day) =

• Lone traveller day trip expenditure = $95/day x 0.657 = $62.39/day +
• Lone traveller overnight trip expenditure = $170/day x 0.073 = $12.41/day +
• Pair traveller day trip expenditure = $59/day x 0.108 = $6.37/day +
• Pair traveller overnight trip expenditure = $84/day x 0.054 = $4.54/day +
• 2-5 group traveller day trip expenditure = $86.75/day x 0.027 = $2.34/day +
• 2-5 group traveller overnight trip expenditure = $124.25/day x 0.081 = $10.06/day

Per-visit-day activity-weighted expenditure associated with a representative MDB eBirder in our sample = **$98.11/day**

Per-visit-day activity-weighted expenditure associated with a representative MDB non-eBirder in our sample ($/day) =

• Lone traveller day trip expenditure = $95/day x 0.525 = $49.88/day +
• Lone traveller overnight trip expenditure = $132.5/day x 0.058 = $7.69/day +
• Pair traveller day trip expenditure = $75/day x 0.139 = $10.42/day +
• Pair traveller overnight trip expenditure = $112.5/day x 0.278 = $31.28/day

Per-visit-day activity-weighted expenditure associated with a representative MDB non-eBirder in our sample = **$99.27/day**

Across survey respondents, there is a 69.1% probability that someone who birdwatches in the Murray-Darling Basin is also an eBird user, a 26.5% probability that someone who birdwatches in the Murray-Darling Basin is not an eBird user, and the remaining 4.4% of respondents are non-MDB birdwatchers (Figure S22, supplemental text section S2). This produces an extrapolation factor of 0.383 (= 26.5/69.1) from eBirder visit days (BuDs) to expected non-eBirder visit days. The per-visit-day activity-weighted expenditures on birdwatching in the Murray-Darling Basin by eBirders and non-eBirders can be applied to the total annual BuDs and the accompanying extrapolated number of non-eBirder visit days at a site to estimate the annual expenditure injected into the local economy from birdwatching visitation (Figure 6A). The same approach can be used to calculate the increase in overall birdwatching expenditure that would be expected to follow from a predicted increase in BuDs following an improvement in ecosystem condition, as proxied by an increase in percentage wet surface area at a site (Figure 6B, Table S9).

##### Birdwatcher interviews

Interviews adopted the well-established oral history methodology of recording life experiences through semi-structured interviews.^53^^,^^54^ The interviews offered testimony of the lived experience, that could challenge (or support) findings from the quantitative analyses.

Interview participants were selected from those who volunteered to be interviewed whilst responding to the online survey. The interviews were designed to record diverse experiential knowledge from a range of birdwatchers throughout the Basin. Interviews provided insights regarding factors that enhanced the birdwatching experience and factors that influenced where people went to watch birds. Respondents were asked to nominate favourite birdwatching locations. This enabled the interviewer to delve deeper into what birdwatchers required and highlight particular birdwatching experiences according to place.

Interviewees comprised a cross section of different genders, from four states and the Australian Capital Territory, who visited a variety of bird watching locations and habitats across the Basin, with varying travel experiences and patterns. People with a range of expenditures on birdwatching were selected. Interviews were conducted via telephone. Sixteen birdwatchers were interviewed.

Interviews were conversational in tone and covered a series of open-ended questions, whilst also moving beyond the questions to explain and enlarge on initial responses, adding depth to the discussion.^55^ A clear project description, methodology and guiding questions provided consistency and transferability across the interviews, thus enabling comparative analysis to extract key insights. Interview starting point questions were as follows:

• What is the attraction of birdwatching for you?
• Where are your favourite birdwatching spots in the Basin and what makes those places special?
• What makes a really good birdwatching trip for you?
• What helps you choose where to go – type of birds, quantity of birds, location, facilities (e.g., bird hide), environment, ease of travel, accommodation, cost?
• What influences the timing of your trips – type of birds, seasons, weather, school holidays?
• How long are your usual trips?
• Do you go alone or in a group and what influences that decision?
• What do you spend money on when travelling for birdwatching?
• How do you know where to go – friends’ recommendation, birdwatching group recommendation, eBird, social media, word of mouth?

### Quantification and statistical analysis

Hotspot-specific and panel data models were implemented in STATA/SE v15.1.^42^ Hotspot-specific models were estimated using STATA’s glm routine with Newey-West Heteroskedasticity and AutoCorrelation robust (HAC) standard errors to ensure robust statistical inference.^50^ Panel data models were estimated using STATA’s xtpoisson fixed effects routine using robust bootstrapped standard errors with bootstrap replications clustered by hotspot. Statistical significance of individual drivers at p < 0.05 and 0.05 ≤ p < 0.10 levels are flagged in yellow and orange, respectively, in tables of regression results.

Online survey data were collated and analysed in Microsoft Excel to obtain relevant descriptive statistics for expenditure calculations. Transcripts from semi-structured interviews were analysed manually to identify key themes which provided qualitative support, in birdwatchers’ own words, for the associations identified in our regression analyses.

## Supplemental information

Document S1. Figures S1–S23 and Tables S1–S4 and S7–S10

Table S5. Unique bird species (and species family groupings) from hotspot sites, related to Results

Table S6. Bird species uitilizing freshwater or freshwater-edge habitats, related to Results

## References

[1] Commonwealth of Australia (2024). Australia’s Strategy for Nature 2024-2030, particularly Objective 5. https://www.dcceew.gov.au/sites/default/files/documents/australias-strategy-for-nature-2024-2030.pdf.

[2] Convention of the Parties to the Convention on Biological Diversity (2022). Global Biodiversity Framework. https://www.cbd.int/doc/c/e6d3/cd1d/daf663719a03902a9b116c34/cop-15-l-25-en.pdf.

[3] Australian Bureau of Statistics (2010). Year Book Australia 2009-10. https://www.abs.gov.au/AUSSTATS/abs@.nsf/Lookup/1301.0Chapter3042009%5f10.

[4] Commonwealth of Australia (2021). Australian Wetlands Database. https://www.dcceew.gov.au/water/wetlands/australian-wetlands-database.

[5] Australian Government (2022). Ramsar wetlands of Australia. https://www.dcceew.gov.au/sites/default/files/documents/ramsar-sites-australia.pdf.

[6] Government of Australia (2012). Basin Plan 2012. https://www.mdba.gov.au/sites/default/files/publications/basin-plan-f2021C01067.pdf.

[7] Murray-Darling Basin Authority (2023). The Murray-Darling Basin Agreement. https://www.mdba.gov.au/water-use/allocations/murray-darling-basin-agreement.

[8] Murray-Darling Basin Authority (2024). Murray-Darling Basin Authority Annual Report 2023-24. https://www.transparency.gov.au/publications/agriculture/murray-darling-basin-authority/murray-darling-basin-authority-annual-report-2023-24.

[9] Australian Bureau of Statistics (2021). Water Account Australia 2020-21. https://www.abs.gov.au/statistics/environment/environmental-management/water-account-australia/2020-21.

[10] Aither (2022). Murray-Darling Basin Social and Economic Conditions Report: a report for the Murray-Darling Basin Authority. https://www.mdba.gov.au/sites/default/files/publications/murray-darling-basin-social-and-economic-conditions-report-2022.pdf.

[11] Murray Regional Tourism (2024). Tourism Australia FY2023-24 International and Domestic Visitor Survey Data. https://www.murrayregionaltourism.com.au/research-resources/murray-research/.

[12] Tardieu L., Tuffery L. (2019). From supply to demand factors: What are the determinants of attractiveness for outdoor recreation?. Ecol. Econ..

[13] Steven R., Morrison C., Arthur J.M., Castley J.G. (2015). Avitourism and Australian important bird and biodiversity areas. PLoS One.

[14] Byrd K.B., Woo I., Hall L., Pindilli E., Moritsch M., Good A., De La Cruz S., Davis M., Nakai G. (2024). Birdwatching preferences reveal synergies and tradeoffs among recreation, carbon, and fisheries ecosystem services in Pacific Northwest estuaries, USA. Ecosyst. Serv..

[15] Whetton P., Chiew F., Hart B.T., Bond N.R., Byron N., Pollino C.A., Stewardson M.J. (2021). Murray-Darling Basin, Australia.

[16] Colloff M., Lavorel S., Wise R.M., Dunlop M., Overton I.C., Williams K.J. (2016). Adaptation services of floodplains and wetlands under transformational climate change. Ecol. Appl..

[17] Manero A., Normyle A., Vardon M., Grafton R.Q. (2024). Two sides of the same coin’? Bridging water accounting and valuation for better decision-making. Environ. Res. Lett..

[18] Australian Bureau of Statistics (2020). Concepts of Tourism. Satellite Accounts: Tourism Satellite Accounts.

[19] Tourism Research Australia (2022). National Visitor Survey: methodology.

[20] Goodchild M.F. (2007). Citizens as sensors: the world of volunteered geography. GeoJournal.

[21] Teles da Mota V., Pickering C. (2020). Using social media to assess nature-based tourism: Current research and future trends. J. Outdoor Recreat. Tour..

[22] Norman P., Pickering C.M., Castley G. (2019). What can volunteered geographic information tell us about the different ways mountain bikers, runners and walkers use urban reserves?. Landsc. Urban Plan.

[23] Kolstoe S., Cameron T.A. (2017). The Non-market Value of Birding Sites and the Marginal Value of Additional Species: Biodiversity in a Random Utility Model of Site Choice by eBird Members. Ecol. Econ..

[24] Guilfoos T., Thomas P., Kolstoe S. (2023). Estimating habit-forming and variety-seeking behavior: Valuation of recreational birdwatching. Am. J. Agric. Econ..

[25] Sinclair M., Ghermandi A., Signorello G., Giuffrida L., De Salvo M. (2022). Valuing Recreation in Italy’s Protected Areas Using Spatial Big Data. Ecol. Econ..

[26] Sullivan B.L., Wood C.L., Iliff M.J., Bonney R.E., Fink D., Kelling S. (2009). eBird: A citizen-based bird observation network in the biological sciences. Biol. Conserv..

[27] Jayalath T.A., Lloyd-Smith P., Becker M. (2023). Biodiversity Benefits of Birdwatching Using Citizen Science Data and Individualized Recreational Demand Models. Environ. Resour. Econ. (Dordr).

[28] Cameron A.C., Trivedi P.K. (2013). Regression analysis of count data.

[29] Long J.S., Freese J. (2014). Regression models for categorical dependent variables using STATA.

[30] Garnett S.T., Duursma D.E., Ehmke G., Guay P.J., Stewart A., Szabo J.K., Weston M.A., Bennett S., Crowley G.M., Drynan D. (2015). Biological, ecological, conservation and legal information for all species and subspecies of Australian bird. Sci. Data.

[31] Ghermandi A. (2022). Geolocated social media data counts as a proxy for recreational visits in natural areas: A meta-analysis. J. Environ. Manage.

[32] Smart J.C.R., Harte J. (2024). Recreational and tourism value of healthy rivers: Extension A – Final Report. https://www.griffith.edu.au/australian-rivers-institute.

[33] Randler C., Staller N., Kalb N., Tryjanowski P. (2023). Charismatic Species and Birdwatching: Advanced Birders Prefer Small, Shy, Dull, and Rare Species. Anthrozoos.

[34] Tryjanowski P., Jankowiak Ł., Mikula P., Czechowski P., Menzel A., Polakowski M. (2024). What factors affect the ‘flocking’ of birdwatchers during bird rarity observations?. People and Nature.

[35] Steven R., Smart J.C.R., Morrison C., Castley J.G. (2017). Using a choice experiment and birder preferences to guide bird-conservation funding. Conserv. Biol..

[36] Steven R. Bird and Nature Tourism in Australia. KBAs in Danger Case Study Report. https://researchportal.murdoch.edu.au/esploro/outputs/report/Bird-and-Nature-Tourism-in-Australia/991005542577807891.

[37] Sánchez-Rivero M., Sánchez-Martín J.M., Rangel M.C.R. (2020). Characterization of birdwatching demand using a logit approach: Comparative analysis of source markets (National vs Foreign). Animals.

[38] Australian Government (2018). Birds Australia - Important Bird Areas (IBA).

[39] Schwoerer T., Dawson N.G. (2022). Small sight—Big might: Economic impact of bird tourism shows opportunities for rural communities and biodiversity conservation. PLoS One.

[40] Sexton-McGrath K. (2025). Birdwatching and twitchers pump billions into Australia’s tourism industry.

[41] Randler C., Jokimäki J., de Salvo M., de Almeida Barbosa R., Staller N., Tryjanowski P., Kaisanlahti-Jokimäki M.-L., Tsai J.-S., Ortiz-Pulido R., Rahafar A. (2023). Spatial, temporal, and motivational changes due to the COVID-19 pandemic in a nature-based leisure activity - A global survey of birders. iScience.

[42] Stata Corp (2017). STATA/SE® v15.1. http://www.stata.com.

[43] Krause C.E., Newey V., Alger M.J., Lymburner L. (2021). Mapping and Monitoring the Multi-Decadal Dynamics of Australia’s Open Waterbodies using Landsat. Remote Sensing (Basel).

[44] Dunn B., Krause C., Newey V., Lymburner L., Alger M.J., Adams C., Yuan F., Ma S., Barzinpour A., Ayers D., McKenna C., Schenk L. (2024). Digital Earth Australia Waterbodies Version 3. Commonwealth of Australia.

[45] Mueller N., Lewis A., Roberts D., Ring S., Melrose R., Sixsmith J., Lymburner L., McIntyre A., Tan P., Curnow S. (2016). Water observations from space: Mapping surface water from 25years of Landsat imagery across Australia. Remote Sens Environ.

[46] Zuur A.F., Ieno E.N., Saveliev A.A. (2017).

[47] Montgomery D.C., Peck E.A. (1992). Introduction to Linear Regression Analysis.

[48] Guimarães P. (2008). The fixed effects negative binomial model revisited. Econ Lett.

[49] Wooldridge J.M. (1999). Distribution-free estimation of some nonlinear panel data models. J. Econom..

[50] Newey W.K., West K.D. (1987). A Simple, Positive Semi-Definite, Heteroskedasticity and Autocorrelation Consistent Covariance Matrix. Econometrica.

[51] Survey Monkey http://www.surveymonkey.com.

[52] Steven R. (2023). https://researchportal.murdoch.edu.au/esploro/fulltext/report/Developing-Bird-Tourism-in-the-Lake/991005712370307891?repId=12169077740007891&mId=13169066730007891&institution=61MUN_INST.

[53] Puri A., Thompson A. (2017). Australian lives: an intimate history.

[54] Perks R., Thompson A. (2015). The Oral History Reader.

[55] Mahuika N. (2019). Rethinking oral history and tradition: An Indigenous perspective.

