# Supplementary material for: An interdisciplinary analysis of recreational birdwatching and wetland extent in the Murray-Darling Basin, Australia
Source: iScience. 2026 Jan 19;29(2):114743. doi: 10.1016/j.isci.2026.114743 (PMC12919282; doi:10.1016/j.isci.2026.114743)
Supplement: Document S1. Figures S1–S23 and Tables S1–S4 and S7–S10 [file mmc1.pdf]

iScience, Volume *29*

Supplemental information

An interdisciplinary analysis of recreational birdwatching and wetland extent in the Murray-Darling Basin, Australia

James C.R. Smart, Jeremy Harte, Margaret Cook, Syezlin Hasan, J. Guy Castley, and Alexandre Lima de F. Teixeira

Document S1

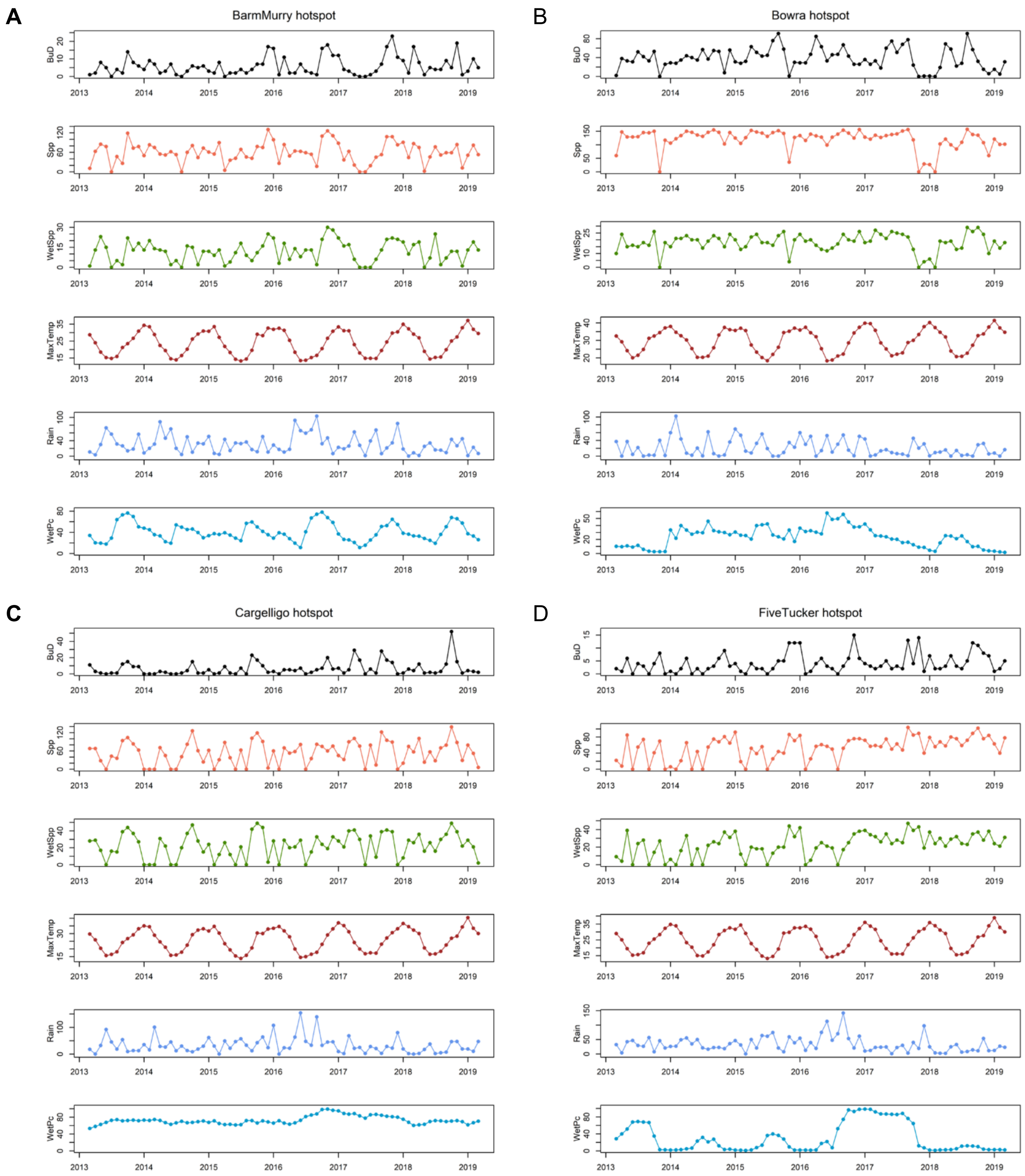

Figure S1. Monthly variation in hotspot-specific birdwatcher visitation, bird species richness and environmental and ecological characteristics for hotspots (A) Barmah Murray Valley National Parks, (B) Bowra Wildlife Reserve, (C) Lake Cargelligo, and (D) Fivebough Swamp and Tuckerbill Wetland. Related to Figure 1B. Monthly variation in birdwatcher visitation (proxied by eBird BuDs) [black], eBird-reported species richness for all birds [orange] and water and water-edge habitat-related bird species [green], average daily maximum temperature [dark red], total rainfall [light blue], and percentage of maximum observed wet surface area for on-site waterbodies [turquoise]..

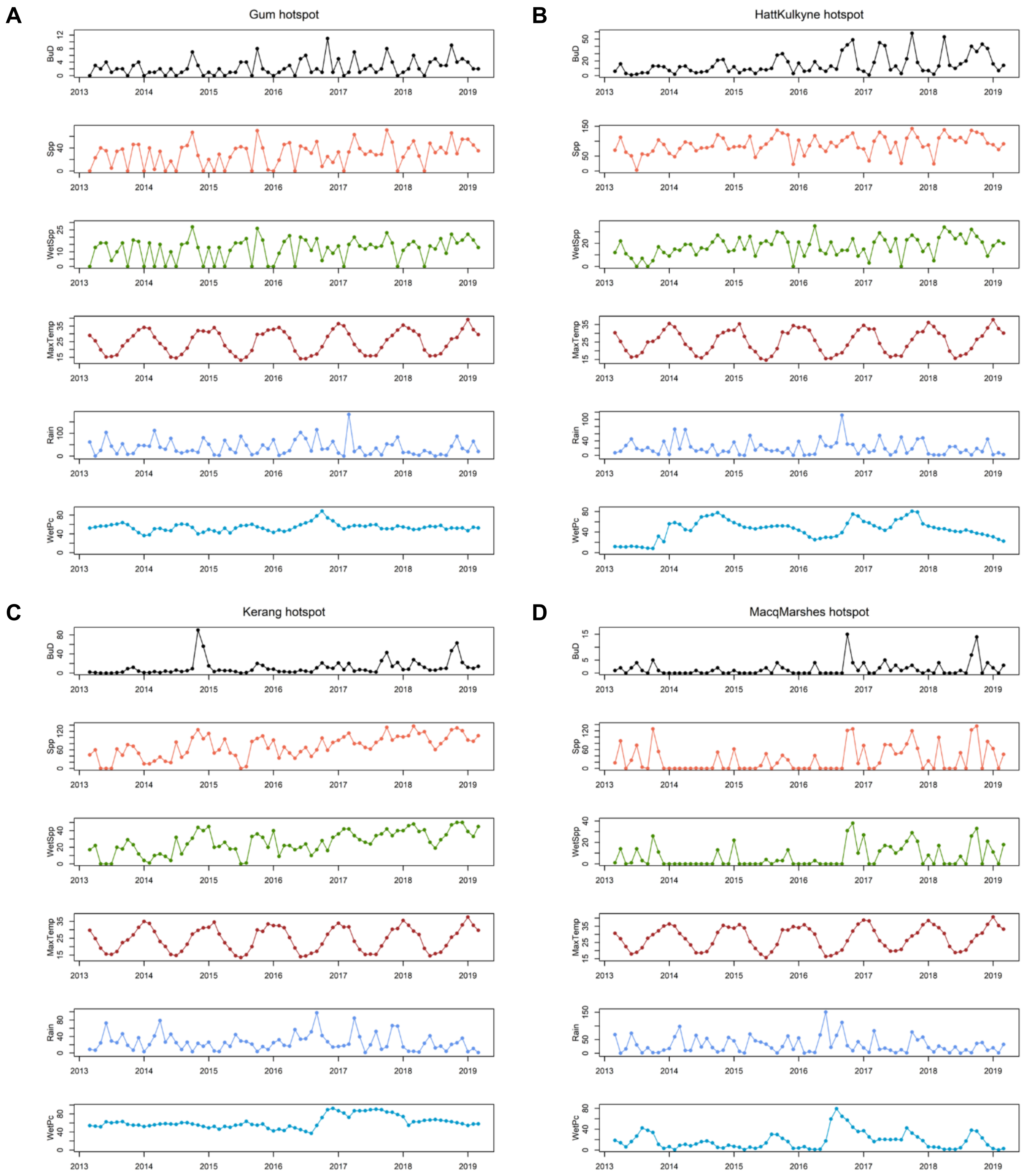

Figure S2 Monthly variation in hotspot-specific birdwatcher visitation, bird species richness and environmental and ecological characteristics for hotspots (A) Gum Swamp (Forbes), (B) Hattah-Kulkyne National Park, (C) Kernang Wetlands, and (D) Macquarie Marshes Nature Reserve. Related to Figure 1B. Monthly variation in birdwatcher visitation (proxied by eBird BuDs) [black], eBird-reported species richness for all birds [orange] and water and water-edge habitat-related bird species [green], average daily maximum temperature [dark red], total rainfall [light blue], and percentage of maximum observed wet surface area for on-site waterbodies [turquoise].

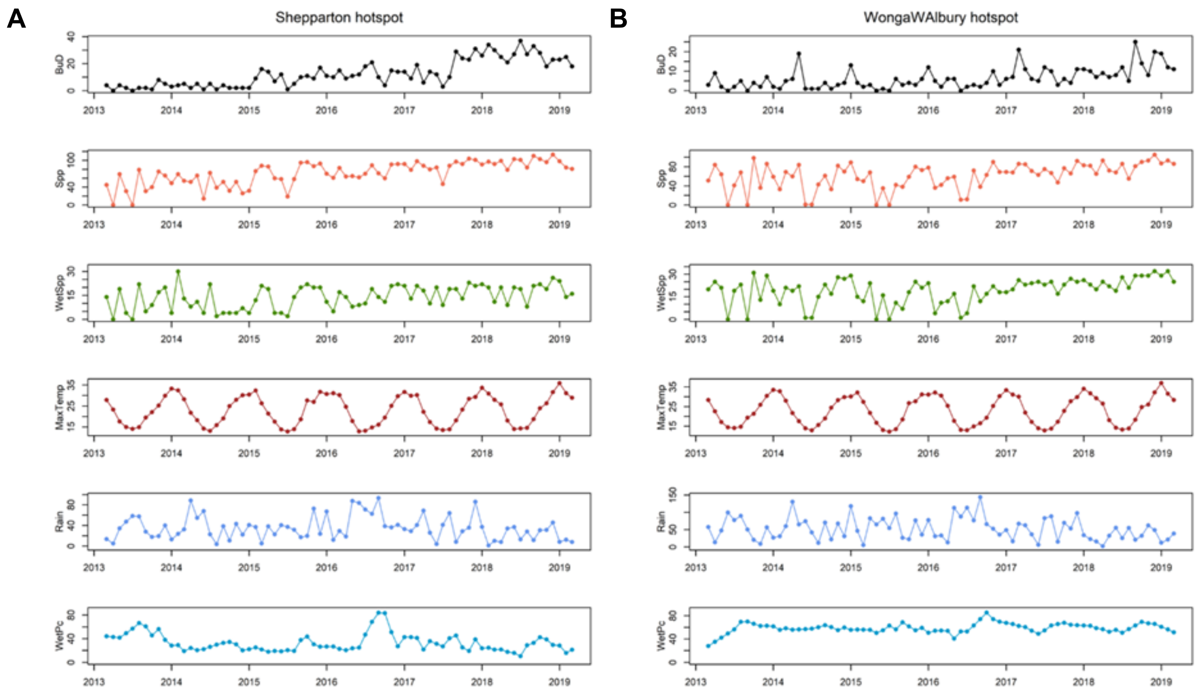

Figure S3 Monthly variation in hotspot-specific birdwatcher visitation, bird species richness and environmental and ecological characteristics for hotspots (A) Shepparton, and (B) Wonga West Albury Wetlands. Related to Figure 1B. Monthly variation in birdwatcher visitation (proxied by eBird BuDs) [black], eBird-reported species richness for all birds [orange] and water and water-edge habitat-related bird species [green], average daily maximum temperature [dark red], total rainfall [light blue], and percentage of maximum observed wet surface area for on-site waterbodies [turquoise].

Table S1. Descriptive statistics of hotspot-specific BuD counts, species list posts and visitation, related to Figure 2A.

Barmah Murray							
eBirders		Species Lists		Days with visits		BuDs	
248		715		299		443	
	min	25%ile	median	mean	75%ile	95%ile	max
Species Lists per eBirder	1	1	2	2.9	3	9	34
Species per species list	1	10	22	22.8	33	51	80
Visit days per eBirder	1	1	1	1.8	2	4	22
BuDs per visit day	1	1	1	1.48	2	3	9

Bowra							
eBirders		Species Lists		Days with visits		BuDs	
282		3825		1753		2821	
	min	25%ile	median	mean	75%ile	95%ile	max
Species Lists per eBirder	1	1.25	4	13.6	8	30.9	1623
Species per species list	1	17	43	43.7	69	91	111
Visit days per eBirder	1	1	3	10	5	14.9	1511
BuDs per visit day	1	1	1	1.61	2	4	10

Cargelligo							
eBirders		Species Lists		Days with visits		BuDs	
194		694		280		476	
	min	25%ile	median	mean	75%ile	95%ile	max
Species Lists per eBirder	1	1	2	3.6	4	11.4	23
Species per species list	1	10	22	24.0	36	53	77
Visit days per eBirder	1	1	2	2.5	3	7	18
BuDs per visit day	1	1	1	1.7	2	4	7

Fivebough Tuckerbill							
eBirders		Species Lists		Days with visits		BuDs	
175		334		222		300	
	min	25%ile	median	mean	75%ile	95%ile	max
Species Lists per eBirder	1	1	1	1.9	2	6	12
Species per species list	1	18	32	31.3	43.8	60.4	75
Visit days per eBirder	1	1	1	1.7	2	4.6	11
BuDs per visit day	1	1	1	1.35	1	3	6

Gum							
eBirders		Species Lists		Days with visits		BuDs	
127		183		140		183	
	min	25%ile	median	mean	75%ile	95%ile	max
Species Lists per eBirder	1	1	1	1.4	2	3	7
Species per species list	1	16	25	24.4	33	43.9	60
Visit days per eBirder	1	1	1	1.4	2	3	7
BuDs per visit day	1	1	1	1.31	1	3	6

Table S1 contd. Descriptive statistics of hotspot-specific BuD counts, species list posts and visitation, related to Figure 2A.

Hattah Kulkyne							
eBirders 439		Species Lists 2247		Days with visits 658		BuDs 1116	
	min	25%ile	median	mean	75%ile	95%ile	max
Species Lists per eBirder	1	1	3	5.1	5	16	70
Species per species list	1	5	12	17.0	24	51	90
Visit days per eBirder	1	1	2	2.5	3	8	24
BuDs per visit day	1	1	1	1.7	2	4	11

Kerang							
eBirders 292		Species Lists 1378		Days with visits 460		BuDs 830	
	min	25%ile	median	mean	75%ile	95%ile	max
Species Lists per eBirder	1	1	2	4.7	4	15	158
Species per species list	1	8.	17	18.9	28	45	85
Visit days per eBirder	1	1	1	2.8	3	7	58
BuDs per visit day	1	1	1	1.8	2	5	11

Macquarie Marshes							
eBirders 48		Species Lists 366		Days with visits 88		BuDs 112	
	min	25%ile	median	mean	75%ile	95%ile	max
Species Lists per eBirder	1	1	3	7.6	7.5	19.6	103
Species per species list	1	7	15	18.0	26	43	108
Visit days per eBirder	1	1	2	2.3	3	6	10
BuDs per visit day	1	1	1	1.27	1	2	6

Shepparton							
eBirders 80		Species Lists 979		Days with visits 739		BuDs 912	
	min	25%ile	median	mean	75%ile	95%ile	max
Species Lists per eBirder	1	1	1	12.2	2	11.9	659
Species per species list	1	27	37	34.1	44	51	69
Visit days per eBirder	1	1	1	11.4	2	9.9	645
BuDs per visit day	1	1	1	1.23	1	2	6

Wonga W Albury							
eBirders 171		Species Lists 542		Days with visits 384		BuDs 478	
	min	25%ile	median	mean	75%ile	95%ile	max
Species Lists per eBirder	1	1	1	3.2	2	8	67
Species per species list	1	9	26	25.3	39.8	52	88
Visit days per eBirder	1	1	1	2.8	2	6	61
BuDs per visit day	1	1	1	1.24	1	2	6

Table S2. Descriptive statistics of hotspot-specific monthly time varying data, related to Figure 1B.

Hotspot	Name	BuD count: monthly total (count)					
		Obs.	Mean	Std. Dev	Median	Min	Max
1	Barmah Murray	72	6	5.4	4	0	23
2	Bowra	72	38.8	22.3	35	0	91
3	Cargelligo	72	6.4	8.7	3	0	52
4	Five Tucker	72	4.2	3.7	3	0	15
5	Gum	72	2.5	2.4	2	0	11
6	Hattah Kulkyne	72	15.5	13.7	12	1	58
7	Kerang	72	11.5	15.5	6	0	90
8	Macquarie Marshes	72	1.6	2.7	0.5	0	15
9	Shepparton	72	12.7	9.9	10.5	0	37
10	Wonga W Albury	72	6.4	5.4	5	0	25
Hotspot	Name	Rainfall: monthly total (mm)					
		Obs.	Mean	Std. Dev	Median	Min	Max
1	Barmah Murray	72	31.6	24.2	26.6	0.1	103.3
2	Bowra	72	20.6	22.1	13.1	0	102.4
3	Cargelligo	72	33.3	30.7	25.6	0	154.3
4	Five Tucker	72	33.5	26.4	25.5	0	142.1
5	Gum	72	37.7	34.6	27.5	0	182.7
6	Hattah Kulkyne	72	21	20.7	14.8	0	111.7
7	Kerang	72	26.2	21	22.5	1	97.8
8	Macquarie Marshes	72	29.3	30.1	19.6	0	150.8
9	Shepparton	72	35.2	23	32.6	1.2	93.5
10	Wonga W Albury	72	52.6	32.5	49.7	2	144.2
Hotspot	Name	Maximum temperature: monthly average of daily max temp. (ºC)					
		Obs.	Mean	Std. Dev	Median	Min	Max
1	Barmah Murray	72	23.9	7.1	24.3	13.3	37.2
2	Bowra	72	29.9	6.8	31.6	18.3	41.4
3	Cargelligo	72	25.7	7.2	26.9	13.8	40.4
4	Five Tucker	72	24.9	7.3	25.9	13.3	39
5	Gum	72	24.9	7.3	26	13.1	39.2
6	Hattah Kulkyne	72	25.3	6.7	25.7	14.7	37.8
7	Kerang	72	24.4	7	25	13.6	37.6
8	Macquarie Marshes	72	27.9	7	29.2	15.7	40.7
9	Shepparton	72	22.9	6.9	23.3	12.8	35.9
10	Wonga W Albury	72	22.9	7.3	22.8	12.2	37

Table S2 contd. Descriptive statistics of hotspot-specific monthly time varying data, related to Figure 1B.

Hotspot	Name	Wet surface area: percentage of max. observed (%)					
		Obs.	Mean	Std. Dev	Median	Min	Max
1	Barmah Murray	72	40.7	17.1	36.7	11.1	78.2
2	Bowra	72	23.7	14.3	25	1.5	57.8
3	Cargelligo	72	72.9	9.5	71.3	57.9	99.3
4	Five Tucker	72	29.8	33.8	11.5	0.9	98.6
5	Gum	72	54.1	8.6	53.5	36.2	88.5
6	Hattah Kulkyne	72	45.9	18.4	48	8.2	80.6
7	Kerang	72	62	13.7	57.8	36.9	92.5
8	Macquarie Marshes	72	17.6	16.9	13.6	0.2	79.3
9	Shepparton	72	33	15.2	28.4	10.4	84.1
10	Wonga W Albury	72	59.3	7.8	58.7	35.3	85.4
Hotspot	Name	Bird species richness via eBird: monthly total, all species (count)					
		Obs.	Mean	Std. Dev	Median	Min	Max
1	Barmah Murray	72	60.4	30.9	59.5	0	130
2	Bowra	72	120.6	37.1	130.5	0	157
3	Cargelligo	72	53.7	37.5	59	0	139
4	Five Tucker	72	53.2	29.2	57.5	0	104
5	Gum	72	30	19.9	33	0	71
6	Hattah Kulkyne	72	88.5	30.1	89.5	3	142
7	Kerang	72	71.9	35.9	78.5	0	137
8	Macquarie Marshes	72	30	40.8	0.5	0	135
9	Shepparton	72	71.9	26.3	79	0	113
10	Wonga W Albury	72	61	27.1	68	0	105
Hotspot	Name	Bird species richness via eBird: monthly total, water species (count)					
		Obs.	Mean	Std. Dev	Median	Min	Max
1	Barmah Murray	72	12	7.7	13	0	30
2	Bowra	72	18.2	6.5	19	0	29
3	Cargelligo	72	22.4	14.7	24	0	49
4	Five Tucker	72	23	12.9	24	0	47
5	Gum	72	12.1	7.4	14	0	27
6	Hattah Kulkyne	72	17.5	8.4	19	0	35
7	Kerang	72	26.1	13.9	26	0	50
8	Macquarie Marshes	72	7.2	10.2	0	0	38
9	Shepparton	72	14	7.3	14	0	30
10	Wonga W Albury	72	19	8.7	21	0	32

Table S3. Key results from the best-fitting regression model of monthly BuD counts for individual hotspots, related to Figure 3A. Statistically significant drivers at $p < 0.05$ are highlighted in yellow, drivers with significance level $0.05 \leq p < 0.10$ are highlighted in orange.

A

Barmah Murray: count data model for monthly BuD counts					
Model	NegBin				
LogLik.	-180.5		AIC	395.0	
Pseudo-R^2	0.118		BIC	433.7	
**Drivers**	**Coef**	**p-val**	**% Effect of + 1 unit (%)**	**delta**	**% Effect of + delta units (%)**
Rain	-0.0059	0.056	-0.59	+25mm	-13.7
MaxTemp	-0.0560	0.114	-5.45	+2ºC	-10.6
Spp_{t-1}	0.0039	0.106	0.39	+10 spp	4.0

B

Bowra: count data model for monthly BuD counts					
Model	NegBin				
LogLik.	-301.5		AIC	639.0	
Pseudo-R^2	0.092		BIC	680.0	
**Drivers**	**Coef**	**p-val**	**% Effect of + 1 unit (%)**	**delta**	**% Effect of + delta units (%)**
Rain	-0.0009	0.835	-0.09	+25mm	-2.1
MaxTemp	0.0207	0.689	2.09	+2ºC	4.2
$WetPc_{t-1}$	0.0132	<0.001	1.33	+15% wet area	21.9
Spp_{t-1}	0.0060	0.025	0.61	+10 spp	6.2

C

Lake Cargelligo: count data model for monthly BuD counts					
Model	NegBin				
LogLik.	-176.1		AIC	386.1	
Pseudo-R^2	0.156		BIC	424.8	
**Drivers**	**Coef**	**p-val**	**Effect of + 1 unit (%)**	**delta**	**Effect of + delta units (%)**
Rain	0.0026	0.285	0.26	+25mm	6.8
MaxTemp	-0.0201	0.739	-1.99	+2ºC	-3.9
$WetPc_{t-1}$	0.0221	<0.001	2.24	+15% wet area	39.4

D

Fivebough Swamp and Tuckerbill Wetland: count data model for monthly BuD counts					
Model	Poisson				
LogLik.	-152.0		AIC	338.1	
Pseudo-R^2	0.302		BIC	376.8	
**Drivers**	**Coef**	**p-val**	**Effect of + 1 unit (%)**	**delta**	**Effect of + delta units (%)**
Rain	-0.0065	0.153	-0.64	+25mm	-14.9
MaxTemp	-0.0753	0.029	-7.25	+2ºC	-14.0
$WetPc_{t-1}$	0.0008	0.445	0.08	+15% wet area	1.2
Spp_{t-1}	-0.0057	0.119	-0.57	+10 spp	-5.6

Table S3 contd. Key results from the best-fitting regression model of monthly BuD counts for individual hotspots, related to Figure 3A. Statistically significant drivers at $p < 0.05$ are highlighted in yellow, drivers with significance level $0.05 \le p < 0.10$ are highlighted in orange.

E

Gum Swamp: count data model for monthly BuD counts					
Model	Poisson				
LogLik.	-125.7		AIC	283.4	
Pseudo-R^2	0.225		BIC	319.9	
**Drivers**	**Coef**	**p-val**	**Effect of + 1 unit (%)**	**delta**	**Effect of + delta units (%)**
Rain	0.0030	0.222	0.30	+25mm	7.8
MaxTemp	0.1370	0.008	14.69	+2ºC	31.5
$WetPc_{t-1}$	0.0269	<0.001	2.72	+15% wet area	49.6

F

Hattah Kulkyne: count data model for monthly BuD counts					
Model	NegBin				
LogLik.	-221.1		AIC	476.2	
Pseudo-R^2	0.175		BIC	514.9	
**Drivers**	**Coef**	**p-val**	**Effect of + 1 unit (%)**	**delta**	**Effect of + delta unit (%)**
Rain	0.0009	0.626	0.09	+25mm	2.4
MaxTemp	-0.0391	0.161	-3.83	+2ºC	-7.5
$WetPc_{t}$	0.0018	0.459	0.18	+15% wet area	2.8

G

Kerang Wetlands: count data model for monthly BuD counts					
Model	NegBin				
LogLik.	-199.8		AIC	433.6	
Pseudo-R^2	0.204		BIC	472.3	
**Drivers**	**Coef**	**p-val**	**Effect of + 1 unit (%)**	**delta**	**Effect of + delta unit (%)**
Rain	-0.0084	0.006	-0.84	+25mm	-19.0
MaxTemp	-0.1291	<0.001	-12.11	+2ºC	-22.8
Spp_{t-1}	0.0156	<0.001	1.58	+10 spp	16.9

H

Macquarie Marshes: count data model for monthly BuD counts					
Model	Poisson				
LogLik.	-94.8		AIC	221.5	
Pseudo-R^2	0.432		BIC	257.9	
**Drivers**	**Coef**	**p-val**	**Effect of + 1 unit (%)**	**delta**	**Effect of + delta units (%)**
Rain	-0.0217	<0.001	-2.14	+25mm	-41.8
MaxTemp	-0.0188	0.680	-1.86	+2ºC	-3.7
$WetPc_{t-1}$	0.0290	<0.001	2.94	+15% wet area	54.5

Table S3 contd. Key results from the best-fitting regression model of monthly BuD counts for individual hotspots, related to Figure 3A. Statistically significant drivers at $p < 0.05$ are highlighted in yellow, drivers with significance level $0.05 \leq p < 0.10$ are highlighted in orange.

I

Shepparton: count data model for monthly BuD counts					
Model	NegBin				
LogLik.	-209.1		AIC	454.2	
Pseudo-R^2	0.182		BIC	495.2	
**Drivers**	**Coef**	**p-val**	**Effect of + 1 unit (%)**	**delta**	**Effect of + delta units (%)**
Rain	0.0002	0.919	0.02	+25mm	0.4
MaxTemp	-0.0070	0.886	-0.70	+2°C	-1.4
$WetPc_{t-1}$	-0.0074	0.001	-0.74	+15% wet area	-10.5
Spp_{t-1}	0.0071	0.035	0.71	+10 spp	7.4

J

Wonga & West Albury Wetlands: count data model for monthly BuD counts					
Model	NegBin				
LogLik.	-185.5		AIC	405.0	
Pseudo-R^2	0.106		BIC	443.7	
**Drivers**	**Coef**	**p-val**	**Effect of + 1 unit (%)**	**delta**	**Effect of + delta units (%)**
Rain	0.0019	0.391	0.19	+25mm	5.0
MaxTemp	-0.0230	0.547	-2.28	+2°C	-4.5
$WetPc_t$	-0.0090	0.185	-0.90	+15% wet area	-12.7

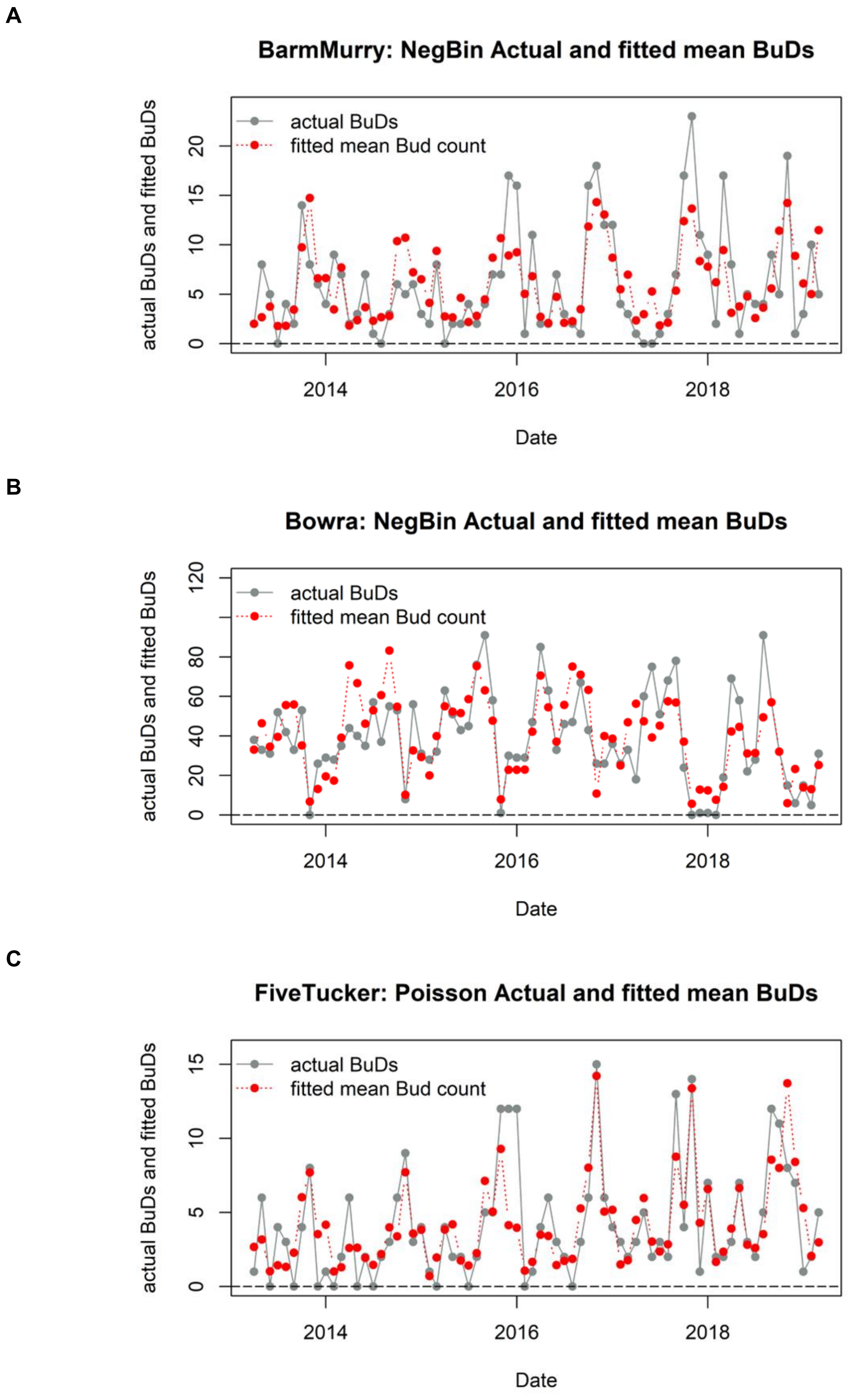

Figure S4. Fitted mean vs. actual monthly BuD counts from the best-fitting negative binomial or Poisson count data models for the (A) Barmah / Murray Valley National Parks, (B) Bowra Wildlife Reserve, and (C) Fivebough Swamp & Tuckerbill Wetland hotspots, related to Figure 3B-C.

A

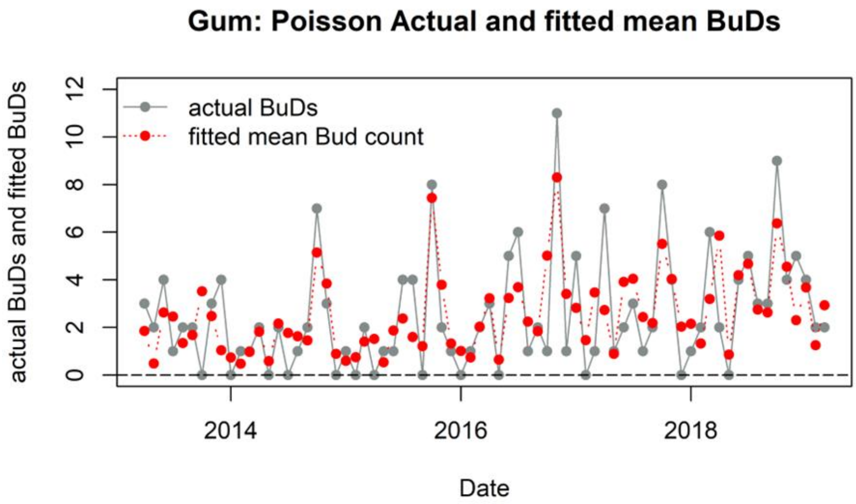

B

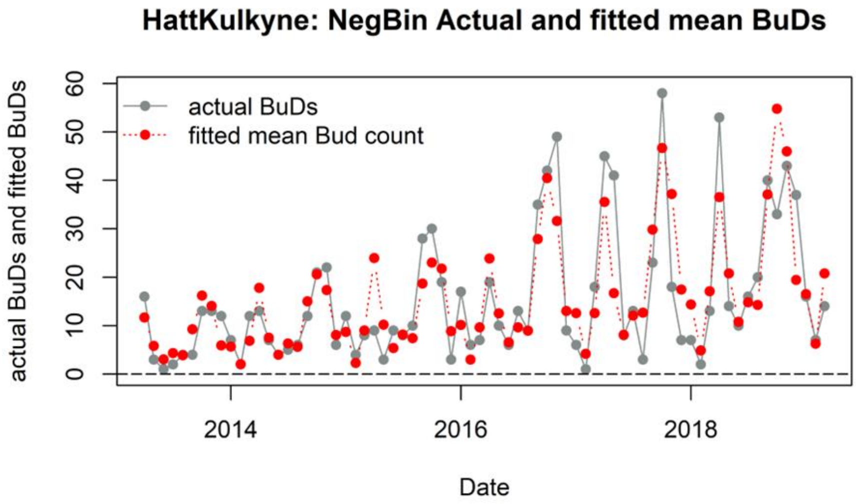

C

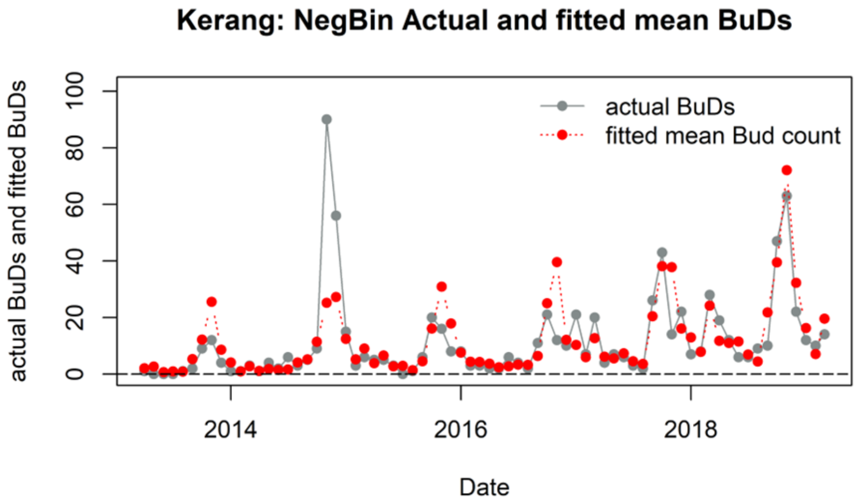

Figure S5 Fitted mean vs. actual monthly BuD counts from the best-fitting negative binomial or Poisson count data models for the (A) Gum Swamp (Forbes), (B) Hattah-Kulkyne National Park, and (C) Kerang Wetlands hotspots, related to Figure 3B-C.

A

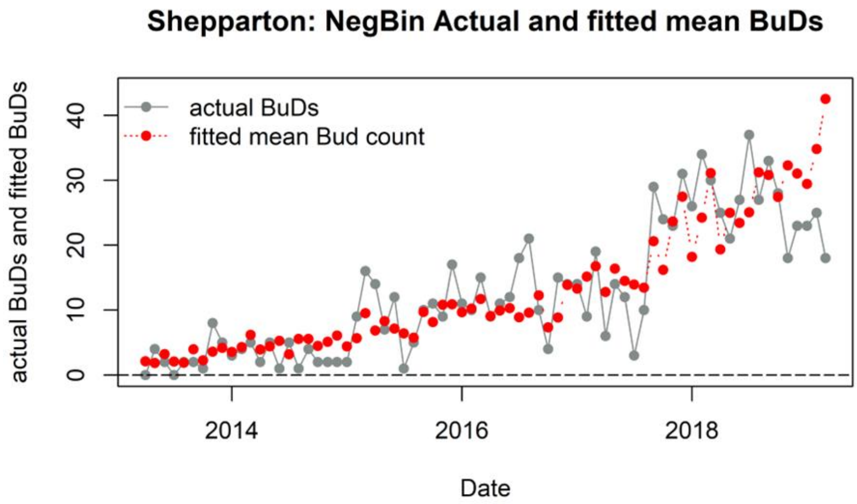

B

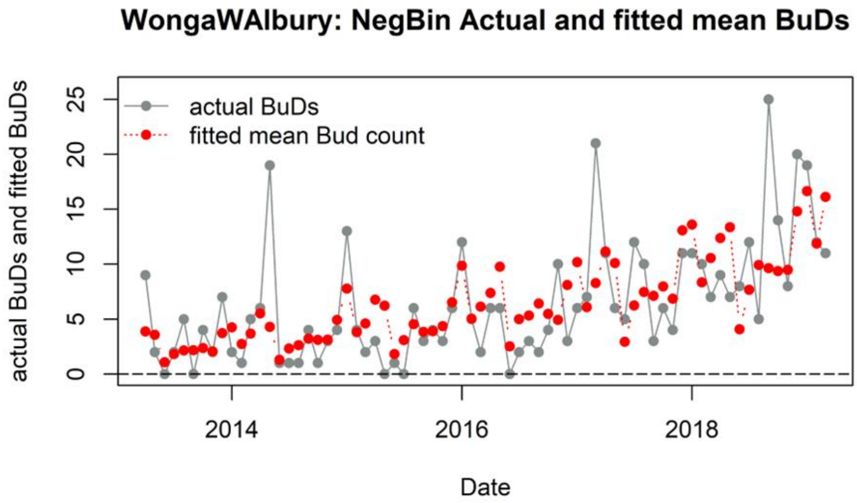

Figure S6 Fitted mean vs. actual monthly BuD counts from the best-fitting negative binomial or Poisson count data models for the (A) Shepparton, and (B) Wonga / West Albury Wetlands hotspots, related to Figure 3B-C.

A

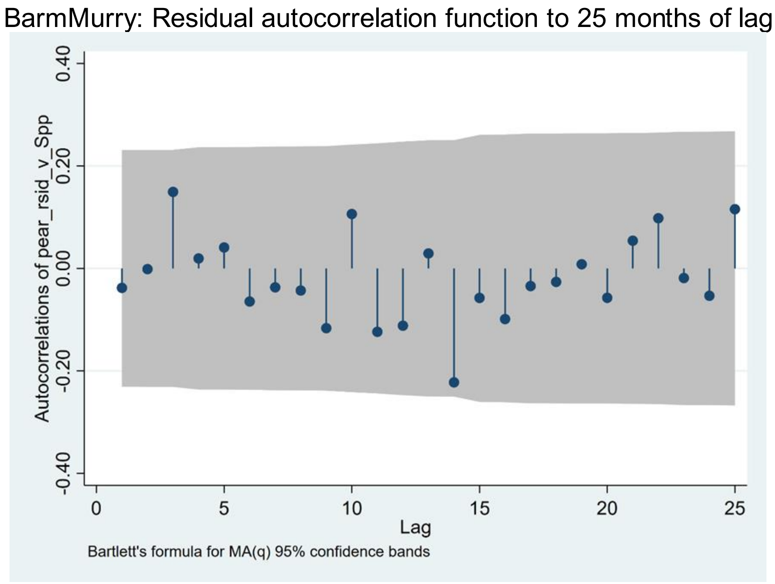

B

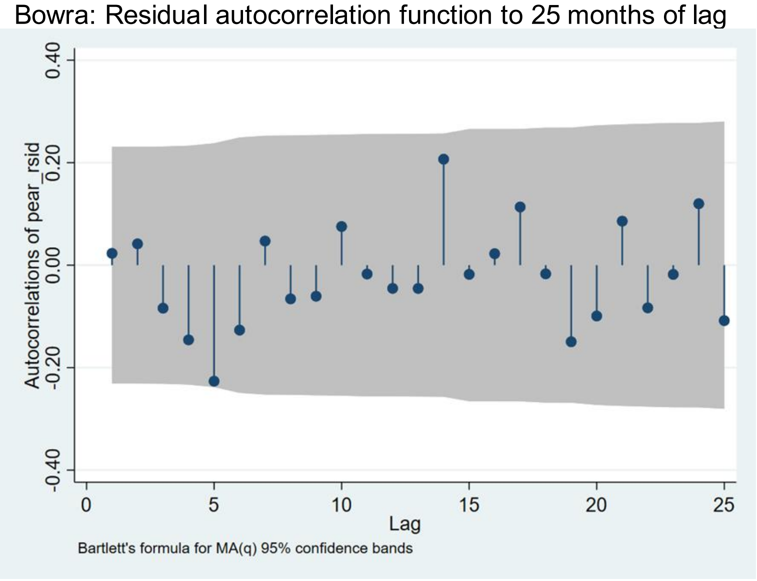

9

C

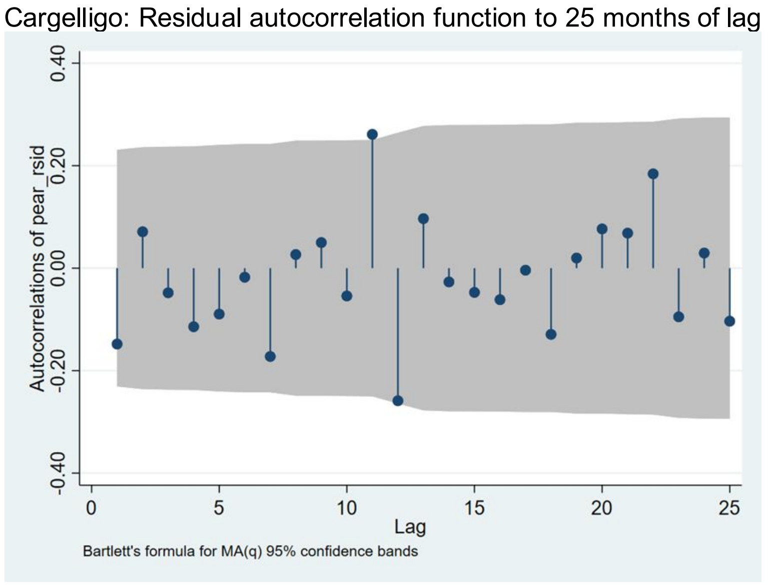

Figure S7. Autocorrelations between the first 25 (monthly) lagged residuals from hotspot-specific models for monthly BuD counts at (A) Barmah / Murray Valley National Parks, (B) Bowra Wildlife Reserve, and (C) Lake Cargelligo, related to Results.

A Fivebough Swamp & Tuckerbill Wetland: Residual autocorrelation function to 25 months of lag

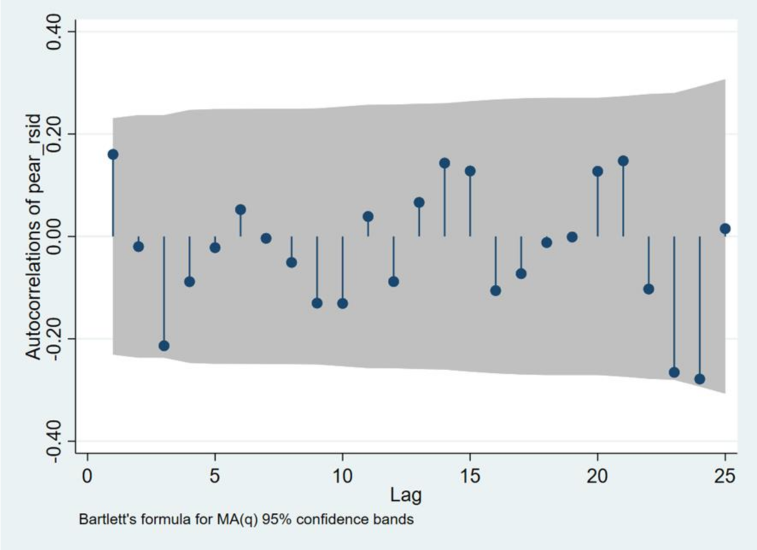

B Gum Swamp (Forbes): Residual autocorrelation function to 25 months of lag

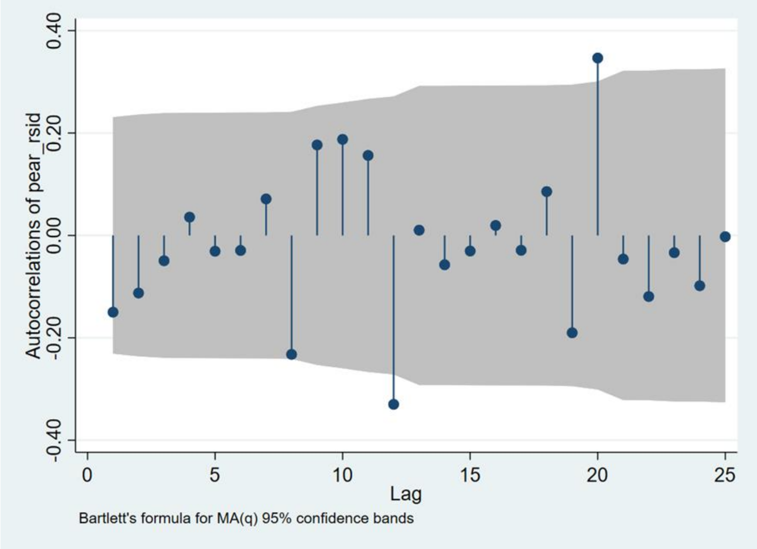

C Hattah-Kulkyne National Park: Residual autocorrelation function to 25 months of lag

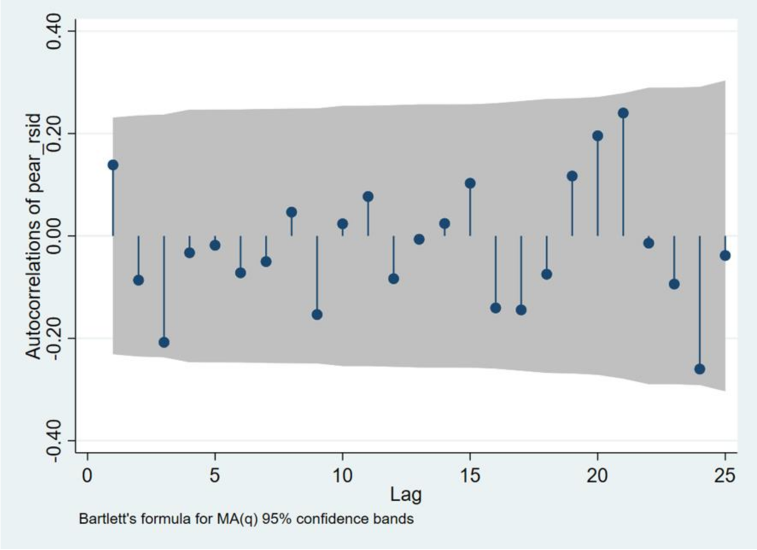

Figure S8 Autocorrelations between the first 25 (monthly) lagged residuals from hotspot-specific models for monthly BuD counts at (A) Fivebough Swamp & Tuckerbill Wetland, (B) Gum Swamp (Forbes), and (C) Hattah-Kulkyne National Park, related to Results.

A Kerang Wetlands: Residual autocorrelation function to 25 months of lag

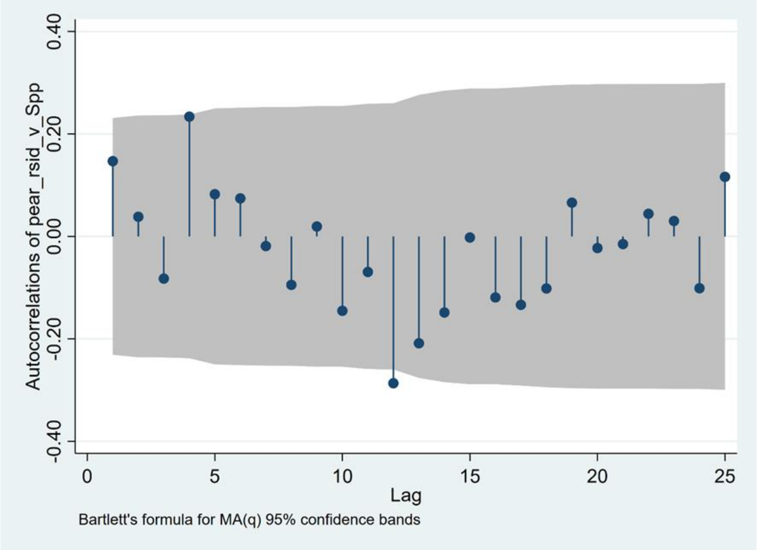

B Macquarie Marshes Nature Reserve: Residual autocorrelation function to 25 months of lag

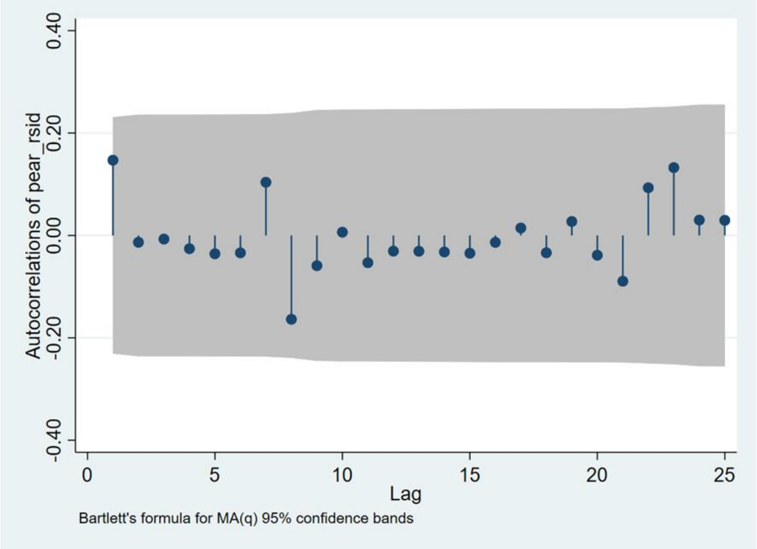

C Shepparton: Residual autocorrelation function to 25 months of lag

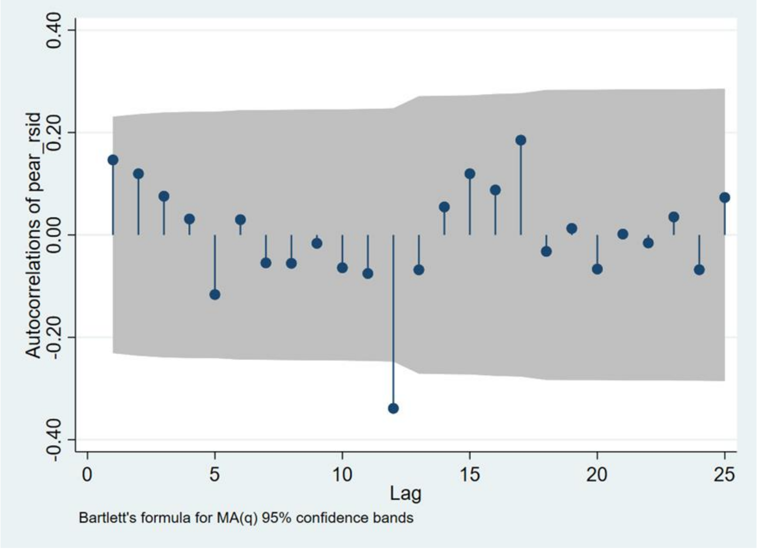

Figure S9. Autocorrelations between the first 25 (monthly) lagged residuals from hotspot-specific models for monthly BuD counts at (A) Kerang Wetlands, (B) Macquarie Marshes Nature Reserve, and (C) Shepparton, related to Results.

Wonga / West Albury Wetlands: Residual autocorrelation function to 25 months of lag

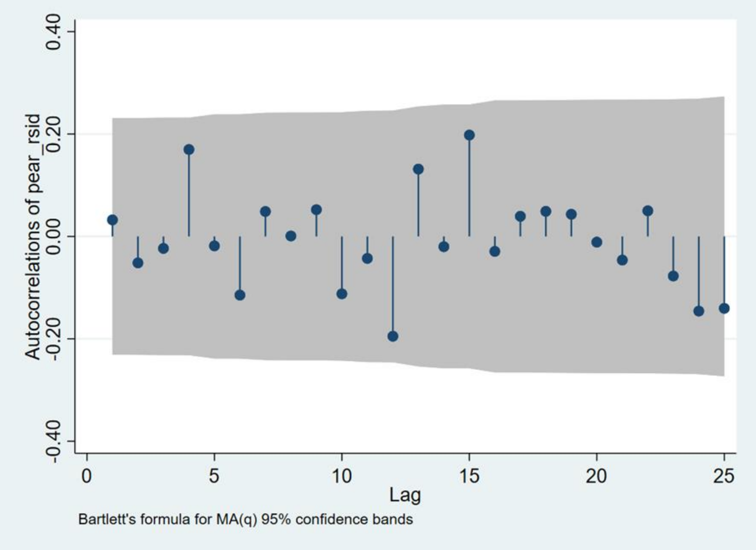

Figure S10 Autocorrelations between the first 25 (monthly) lagged residuals from hotspot-specific models for monthly BuD counts at Wonga / West Albury Wetlands, related to Results.

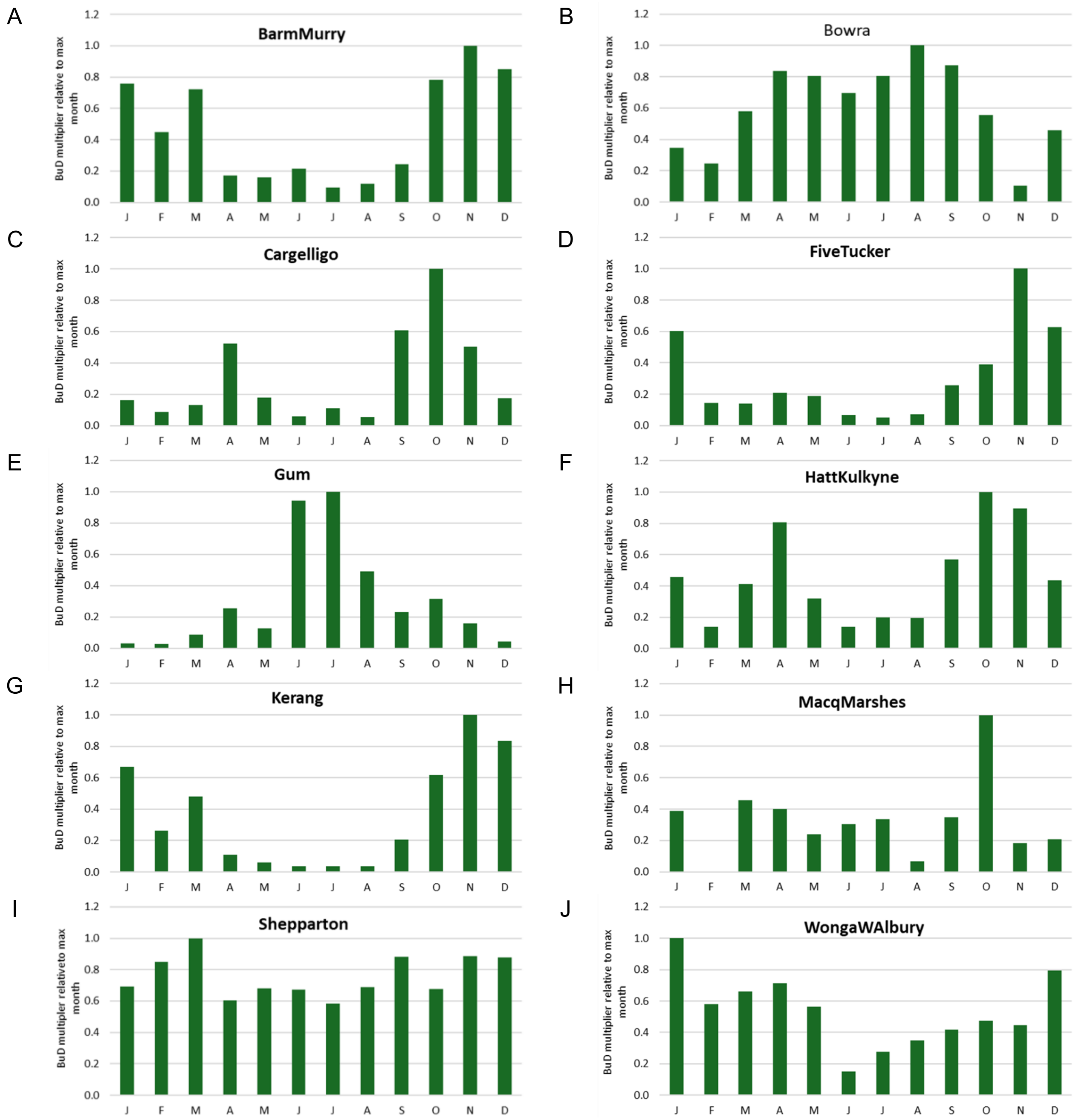

Figure S11. Fitted month-of-the-year terms in individual hotspot regressions on monthly BuD counts, related to Results. (A to J) Monthly BuD multiplier effects normalised to the maximum monthly multiplier effect for each individual hotspot site.

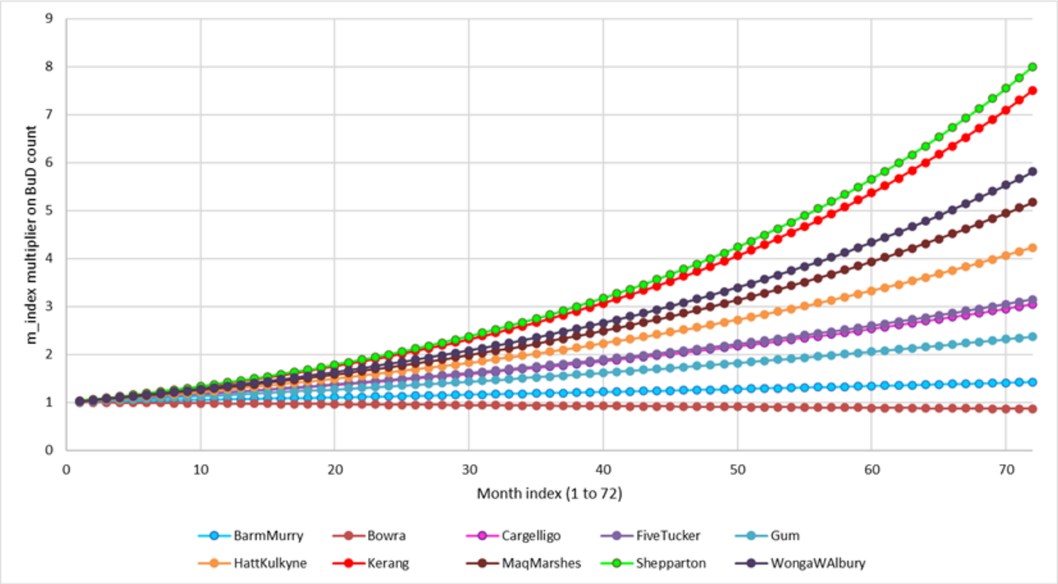

Figure S12. Month-index multiplier time trends for individual hotspots, for month-index 1 (April 2013) to 72 (March 2019), related to Results.

A

Ten-hotspot panel: count data model for monthly BuD counts					
Model	Poisson fixed effects		Robust vce estimation via hotspot cluster bootstrap		
LogLik.	-3009.23			No. obs	720
Wald Chi^2(16)	1521.18			No. groups	10
Prob>Chi^2	<0.001			Grouping by	hotspot
**Drivers**	**Coef**	**p-val**	**Effect of + 1 unit (%)**	**delta**	**Effect of + delta units (%)**
Rain	-0.0033	0.057	-0.33	+25mm	-7.9
MaxTemp	-0.0247	0.178	-2.44	+2°C	-4.8
$WetPc_{t-1}$	0.0043	0.309	0.44	+15% wet area	6.7
Spp_{t-1}	0.0088	<0.001	0.89	+10 spp	9.3

Statistically significant drivers at $p < 0.05$ are highlighted in yellow. Drivers with significance level $0.05 \leq p < 0.10$ are highlighted in orange.

B

Ten-hotspot panel: count data model for bird species richness (all birds)					
Model	Poisson fixed effects		Robust vce estimation via hotspot cluster bootstrap		
LogLik.	-8796.34			No. obs	720
Wald Chi^2(16)	3372.93			No. groups	10
Prob>Chi^2	<0.001			Grouping by	hotspot
**Drivers**	**Coef**	**p-val**	**Effect of + 1 unit (%)**	**delta**	**Effect of + delta units (%)**
Rain	-0.0010	0.220	-0.10	+25mm	-2.4
MaxTemp	0.0109	0.326	1.10	+2°C	2.2
$WetPc_{t-1}$	0.0048	0.040	0.48	+15% wet area	7.4
**Ten-hotspot panel: count data model for bird species richness (water-related birds)**					
Model	Poisson fixed effects		Robust vce estimation via hotspot cluster bootstrap		
LogLik.	-3668.85			No. obs	720
Wald Chi^2(16)	4170.19			No. groups	10
Prob>Chi^2	<0.001			Grouping by	hotspot
**Drivers**	**Coef**	**p-val**	**Effect of + 1 unit (%)**	**delta**	**Effect of + delta units (%)**
Rain	-0.0008	0.314	-0.08	+25mm	-2.0
MaxTemp	0.0174	0.229	1.76	+2°C	3.5
$WetPc_{t-1}$	0.0056	0.003	0.56	+15% wet area	8.8

Statistically significant drivers at $p < 0.05$ are highlighted in yellow. Drivers with significance level $0.05 \leq p < 0.10$ are highlighted in orange.

Figure S13. Regression results from ten-hotspot panel models, related to Results. (A) Results from panel model regression on monthly BuD counts. (B) Results from panel model regressions on reported bird species richness (all birds) and reported species richness of water and water-edge habitat-related birds, when percentage wet area from the *preceding* month is included as a driver.

Ten-hotspot panel: count data model for bird species richness (all birds)					
Model	Poisson fixed effects		Robust vce estimation via hotspot cluster bootstrap		
LogLik.	-8857.54			No. obs	720
Wald $Chi^2(16)$	3967.04			No. groups	10
Prob>Chi^2	<0.001			Grouping by	hotspot
**Drivers**	**Coef**	**p-val**	**Effect of + 1 unit (%)**	**delta**	**Effect of + delta units (%)**
Rain	-0.0012	0.142	-0.12	+25mm	-3.0
MaxTemp	0.0113	0.301	1.14	+2°C	2.3
$WetPc_t$	0.0037	0.091	0.37	+15% wet area	5.7
**Ten-hotspot panel: count data model for bird species richness (water-related birds)**					
Model	Poisson fixed effects		Robust vce estimation via hotspot cluster bootstrap		
LogLik.	-3699.91			No. obs	720
Wald $Chi^2(16)$	2530.22			No. groups	10
Prob>Chi^2	<0.001			Grouping by	hotspot
**Drivers**	**Coef**	**p-val**	**Effect of + 1 unit (%)**	**delta**	**Effect of + delta units (%)**
Rain	-0.0011	0.194	-0.11	+25mm	-2.6
MaxTemp	0.0180	0.198	1.82	+2°C	3.7
$WetPc_t$	0.0040	0.015	0.40	+15% wet area	6.2

Statistically significant drivers at $p < 0.05$ are highlighted in yellow. Drivers with significance level $0.05 \leq p < 0.10$ are highlighted in orange.

Figure S14. Regression results from ten-hotspot panel models, related to Results. Results from panel model regressions on reported bird species richness (all birds) and reported species richness of water and water-edge habitat-related birds, when percentage wet area from the *current* month is included as a driver.

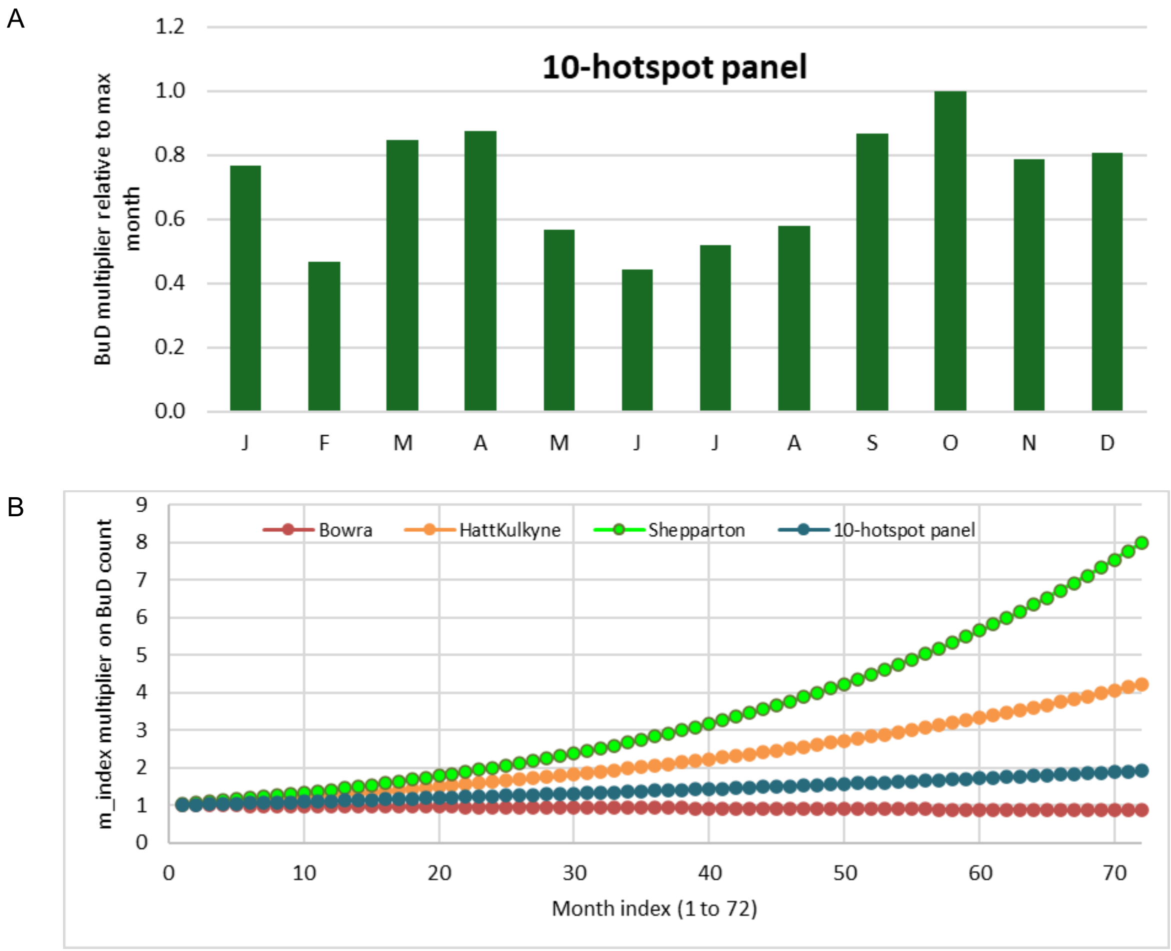

Figure S15. Month-of-the-year and time trend effects from ten-hotspot panel model of monthly BuD counts, related to Results. (A) Month-of-the-year BuD multiplier factors from the 10-hotspot panel model of monthly BuD counts. Multipliers scaled relative to the highest month (October). (B) Month-index time trend from the 10-hotspot panel model of monthly BuD counts. High, mid and low time trends from the individual hotspot models shown for comparison.

A

Hotspot	Pseudo R^2	Linear R^2 equivalent	% effect of +δ change on species richness (all birds)			
			Rain	MaxTemp	$WetPc_{t-1}$	$WetPc_t$
			δ = +25mm	δ = +2°C	δ = +15%	δ = +15%
Barmah Murray	0.019	0.065	ns	ns	ns	
Bowra	0.025	0.081	ns	ns	+17.8	
Cargelligo	0.024	0.078	ns	ns	ns	
Fivebough Tuckerbill	0.019	0.065	ns	ns	+5.5	
Gum	0.013	0.047	ns	+29.3	ns	
Hattah Kulkyne	0.059	0.165	ns	-3.6	ns	
Kerang	0.051	0.146	-17.8	-12.8	+6.2	
Macquarie Marshes	0.056	0.158	-61.9	ns	ns	
Shepparton	0.048	0.139	ns	ns		ns
Wonga W Albury	0.032	0.099	ns	ns	ns	

Grey cells denote that a variable was not included in the best-fitting model for the relevant site. Statistically significant drivers at $p < 0.05$ are highlighted in yellow. Drivers with significance level $0.05 \leq p < 0.10$ are highlighted in orange. ns denotes 'not statistically significant'.

B

Name	Pseudo R^2	Linear R^2 equivalent	% effect of +δ change on species richness (water birds)			
			Rain	MaxTemp	$WetPc_{t-1}$	$WetPc_t$
			δ = +25mm	δ = +2°C	δ = +15%	δ = +15%
Barmah Murray	0.033	0.102	ns	ns	ns	
Bowra	0.046	0.134	ns	ns	+11.6	
Cargelligo	0.034	0.104	ns	+17.5	ns	
Fivebough Tuckerbill	0.031	0.097	ns	ns	+8.0	
Gum	0.019	0.065	+7.7	+28.6	ns	
Hattah Kulkyne	0.075	0.201	-9.8	ns	+13.1	
Kerang	0.074	0.199	-15.8	ns	+10.1	
Macquarie Marshes	0.090	0.234	-51.3	ns	ns	
Shepparton	0.042	0.124	ns	ns	+8.9	
Wonga W Albury	0.045	0.132	ns	ns		-18.6

Grey cells denote that a variable was not included in the best-fitting model for the relevant site. Statistically significant drivers at $p < 0.05$ are highlighted in yellow. Drivers with significance level $0.05 \leq p < 0.10$ are highlighted in orange. ns denotes 'not statistically significant'.

Figure S16. Summary regression results from individual hotspot models for monthly variation in bird species richness, related to Results. (A) Summary results from hotspot-specific count data models of bird species richness (all birds). (B) Summary results from hotspot-specific count data models of bird species richness (water and water-edge habitat-related species).

Table S4. Number of waterbodies listed in Digital Earth Australia's (DEA's) Waterbodies dataset,[S1,S2] total area of DEA waterbodies, number of Bureau of Meteorology (BoM) reporting grid cells, and local government area(s) at each hotspot location, related to Methods.

Hotspot	No. DEA Waterbodies	Total DEA Waterbodies area (m^2)	No. BoM reporting 5.6km x 5.6km gridcells	Local Government Area
Barmah/Murray Valley National Parks selection	411	45,797,400	44	Murray River (59%), Moira (35%), Berrigan (4%), and Edward River (2%)
Bowra Wildlife Sanctuary	34	1,428,300	13	Paroo (100%)
Lake Cargelligo	19	18,964,800	4	Lachlan (81%) and Cobar (19%)
Fivebough Swamp and Tuckerbill Wetland	2	2,672,100	1	Leeton (96%), and Narrandera (4%)
Gum Swamp (Forbes NSW)	14	432,000	1	Forbes (100%)
Hattah Kulkyne National Park	110	21,429,000	28	Mildura (86%), Balranald (8%), Swan Hill (4%), and Wentworth (2%)
Kerang Wetlands	28 of 74	65,124,000 of 72,400,500	17	Gannawarra (96%), Swan Hill (4%)
Macquarie Marshes Nature Reserve	114	6,471,000	41	Warren (64%), Coonamble (35%), and Walgett (1%)
Shepparton	41 of 54	2,987,100 of 4,374,000	2	Greater Shepparton (100%)
Wonga and West Albury Wetlands	91	5,038,200	3	Albury (62%), Wodonga (35%), and Greater Hume Shire (3%)

Table S5. Unique bird species (and species family groupings) listed on eBird species checklists posted from named hotspot sites between 1st April 2013 and 31st March 2019 (inclusive), related to Results. eBird citizen science data were obtained for research use, with authorization, from the Cornell Lab of Ornithology, 159 Sapsucker Woods Road, Ithaca, NY, 14850, USA; eBird@cornell.edu. [S5] The authors particularly thank Jenna Curtis (eBird Project Leader) for helpful comments and suggestions.

Table provided as Excel file: Unique_species_all_sites.xlsx

Table S6. 'Long list' of potential bird species for inclusion in monthly counts of bird species richness for bird species utilising freshwater or freshwater-edge habitats. 'Long list' comprises bird species in the following feeding habitat and breeding habitat categories from Garnett et al. (2015),[S3] related to Results: **Feeding habitats**: Inland waters: Rivers and streams, Deep open waters, Shallow open waters, Reeds and tall wet grassland, Low marshland and wet grassland. **Breeding habitats**: Inland wetland.

Table provided as Excel file: Spp List Waterbirds.xlsx

Table S7. Derived 'home country' for all eBirders posting species lists from Gunbower National Park and Koondrook-Perricoota State Forest between 1 April 2013 and 31 March 2019, related to Discussion. Home country is determined from the country locations of all eBird species lists posted by each individual eBirder in a 2-year window prior to the date on which they posted a bird species list from Gunbower National Park and Koondrook-Perricoota State Forest. 'Home country' analysis from Smart & Harte (2024).[S4]

eBird citizen science data were obtained for research use, with authorization, from the Cornell Lab of Ornithology, 159 Sapsucker Woods Road, Ithaca, NY, 14850, USA; eBird@cornell.edu.[S5] The authors particularly thank Jenna Curtis (eBird Project Leader) for helpful comments and suggestions.

Derived 'home country' for all eBirders posting from Gunbower National Park and Koondrook-Perricoota State Forest between 1st April 2013 and 31st March 2019	
Total number of eBirders posting	82
Total number of posting eBirders with identifiable 'home country'	76
'Home country' of eBirders visiting Gunbower, Perricoota, Koondrook	
Australia	68 (83%)
Identifiable 'home countries' other than Australia	8 (10%)
Undetermined 'home country'	6 (7%)
Species list postings in 2-year window prior to visiting Gunbower, Perricoota, Koondrook	
Total number of eBird species lists posted globally in 2-year window	18,849
Average number of global posts per eBirder in 2-year window	230

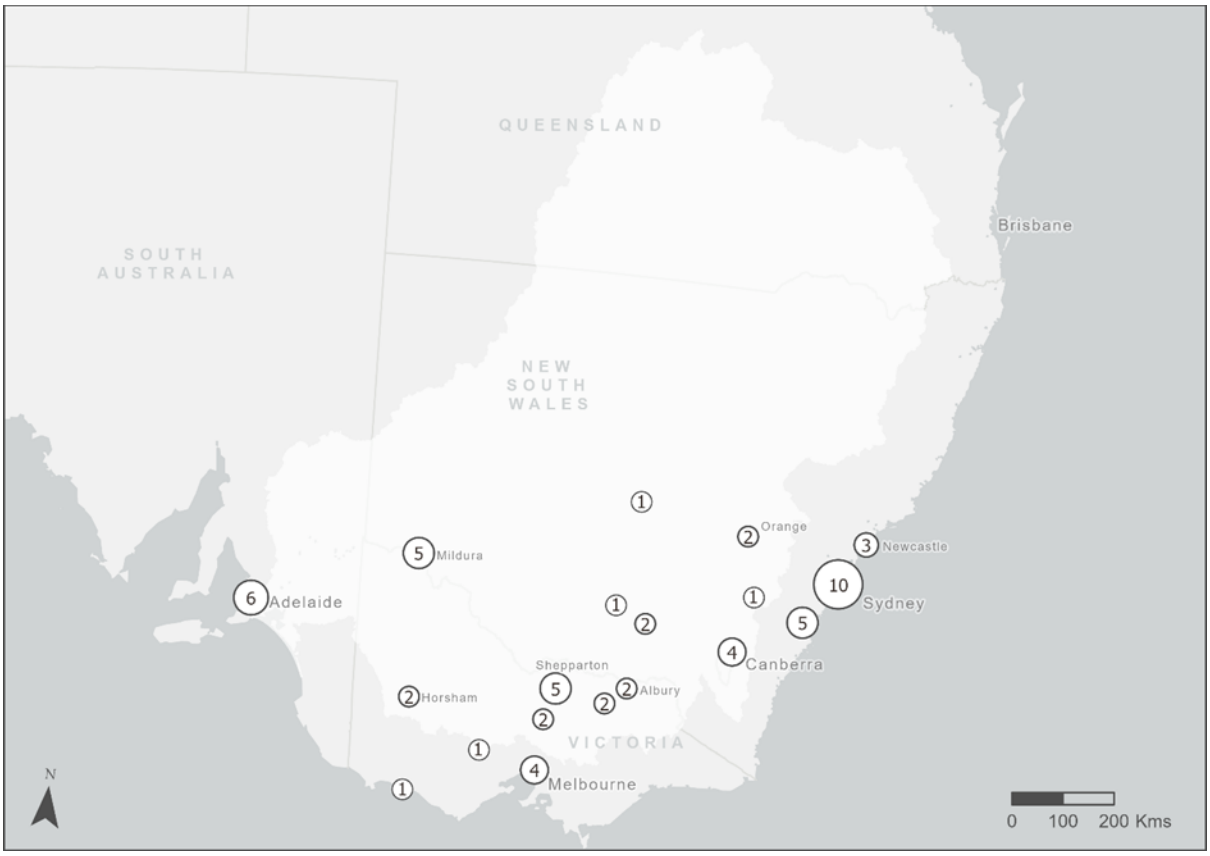

Figure S17. Home locations (identified by postcode) of respondents to the online survey distributed via local birdwatching groups and birdwatching clubs, related to Figure 2B.

Supplemental Text Section S1

Online Birdwatchers' Survey

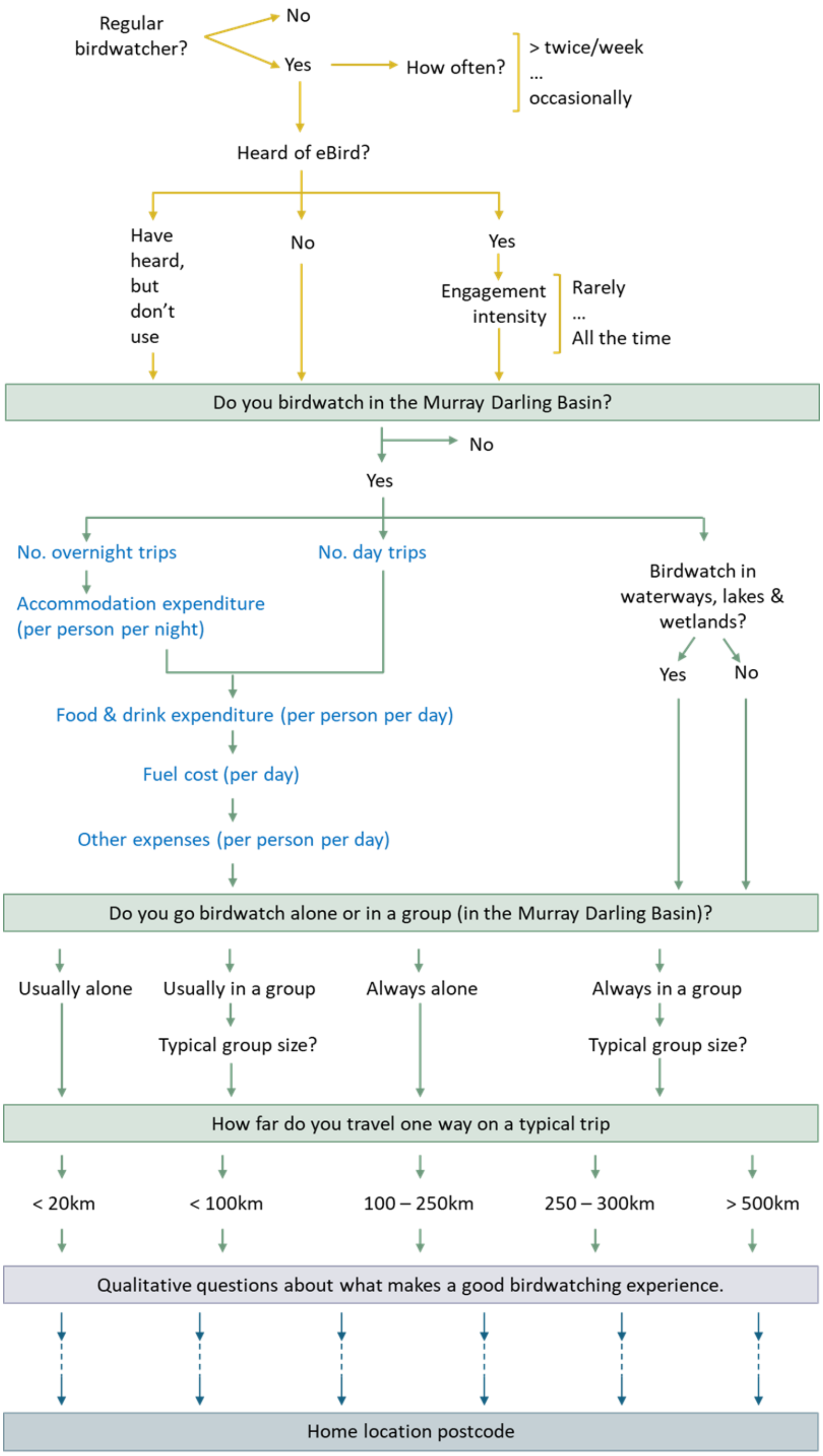

Figure S18. Flow of questions in the online survey distributed via Birdlife Australia Local Groups, related to Figure 2B, Figure 5 and Methods.

The complete set of survey questions can be obtained from the Lead Author on request.

Supplementary Text Section S2

Calculation of per-visit-day activity-weighted trip expenditures from birdwatcher online survey responses

We are interested in estimating the increase in birdwatching-related expenditure that could follow from increased birdwatching visitation at a hotspot site following an improvement in the site's ecosystem health, where ecosystem health improvement is proxied by an increase in wet surface area. To support this analysis, a survey was implemented with members of Birdlife Australia's local birdwatching groups and local birdwatching clubs in New South Wales, South Australia, Victoria and the Australian Capital Territory to obtain information on the number of day and overnight birdwatching trips group members make to the Murray-Darling Basin over a year, whether they typically travel alone or as part of a group, and the typical birdwatching expenditures they incur.

Whilst many birdwatchers use the eBird citizen science app to log their birdwatching activities with differing levels of engagement intensity, some birdwatchers do not use the eBird app at all. The survey therefore elicited levels of engagement with eBird to quantify the proportions of eBird users and non-eBird users among survey respondents. Knowing these relative proportions enables the total number of birdwatching visits (by eBird users and non-eBird users combined) to be estimated by extrapolation from annual eBird BuD counts at hotspot sites. In combination with regression results, this also enables the predicted increase in birdwatcher visitation following an increase in wet surface area at hotspot sites to be estimated.

Key objectives of data collection via the birdwatcher survey were to determine:

1. The proportion of local birdwatching group members who do, or do not, use the eBird citizen science app.

2. If local birdwatching group members go birdwatching at river, lake and wetland sites in the Murray-Darling Basin.

3. If local birdwatching group members typically travel alone or as part of a group (in specified group size categories) on birdwatching trips to the Murray-Darling Basin.

4. The number of day and overnight birdwatching trips made to the Murray-Darling Basin in a typical year.

5. Typical expenditures on day and overnight trips, by expenditure component (food & drink, fuel, accommodation etc.)

Proportion of respondents who use eBird and proportion of respondents who go birdwatching in the Murray-Darling Basin

In response to the question 'Have you heard of the eBird website where you can post your bird sightings online (www.eBird.org)?', 49 respondents (70.6%) answered 'Yes' (i.e., were eBird users) and 20 (29.4%) answered either 'No' or 'Have heard of it but don't use it' (i.e., were not eBird users) (Figure 2B). The proportions of local birdwatching group members (both eBird users and non-eBird users) who birdwatch in the Murray-Darling Basin are determined from responses to the question 'Do you birdwatch on waterways, lakes, wetlands or other types of sites within the Murray-Darling Basin?', 65 respondents answered 'Yes' and 3 answered 'No'. Of the 'Yes' respondents, 47 were eBird users and 18 were not eBird users. Of the 'No' respondents, 1 was an eBird user and 2 were not (Figure 2B).

Numbers travelling alone or as part of a group

We asked respondents whether they travel alone or in a group (Survey question: 'Do you go birdwatching alone or in a group'; four answer choices: 'usually alone', 'usually in a group', 'always alone' and 'always in a group'). Based on the answers given, we determine the proportions of those who travel alone and those who travel in a group (Figure S19, Figure 2B, Figure 5). To gain further

information on the group size for those who travel in a group, we posed a follow-up question 'If in a group, what is the typical group size?' with the following answer options for the group sizes: only 2, 2-5, 6-10, 11-20, and over 20.

Of those who travelled in a group, we determine the proportions of respondents who selected the relevant categories for the group sizes (Figure S19, Figure 2B, Figure 5). Based on 59 responses received to the questions 'Do you go birdwatching alone or in a group?' and 'If in a group, what is the typical group size?', 34 (58% in total), comprising 27 eBirders and 7 non-eBirders, selected the 'usually alone' category and the remaining 25 selected the 'usually in a group' category (Figure S19, Figure 2B, Figure 5). Of those who usually travel in a group, 11, comprising 6 eBriders and 5 non-eBriders, usually travel as a pair and 4 (all of whom were eBirders) usually travel in a group of 2–5 people (Figure S19, Figure 2B, Figure 5). Respondents who birdwatch alone, birdwatch with another person, or birdwatch in a group of between two and five birdwatchers constitute 83% of the 59 respondents to these questions.

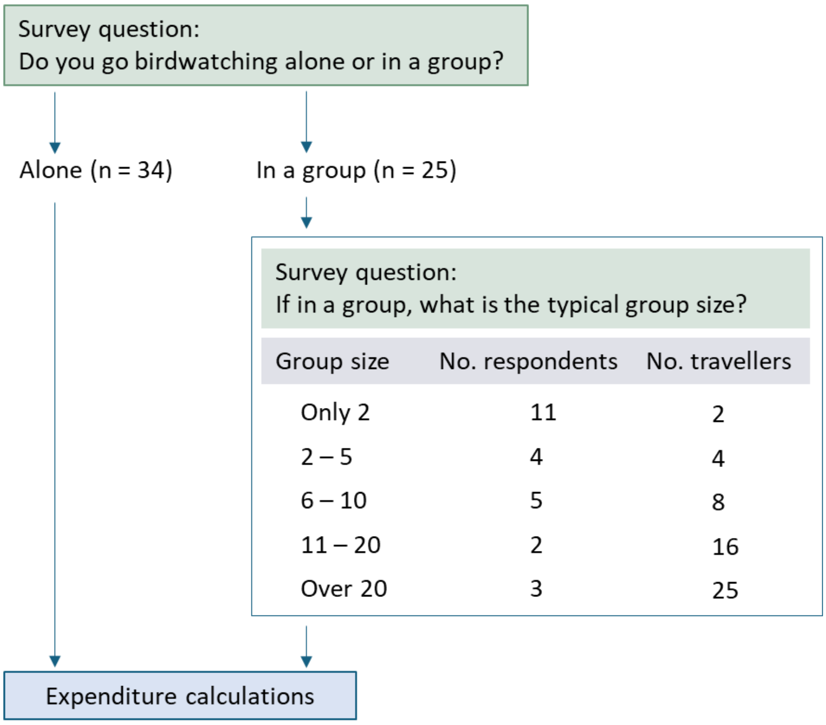

Figure S19. Distribution of group sizes to inform trip expenditure calculations, related to Figure 2B, Figure 5 and Methods.

Responses to 'Always alone' and 'Usually alone' are labelled as 'Alone'. Responses to 'Always in a group' and 'usually in a group' are labelled as 'In a group'. Survey responses were used to determine the proportions of birdwatchers who travel alone and who travel in a group. Additionally, of those who usually travelled in a group, we determine the proportions who travel: as a pair ('only 2' category), in a group of between two and five people ('2-5 ' category), in a group of between six and ten people ('6-10' category), in a group of between eleven and twenty people ('11-20' category), and in a group of more than twenty people ('over 20' category). Numbers in each category are then split again into eBirders and non-eBirders.

Numbers of overnight and day birdwatching trips

Data on the number of overnight trips and day trips undertaken by survey respondents were collected via ‘tick the box’ categories. Tick the box categories were converted into numbers of overnight and day trips per year as shown in Table S8 and Table S9, respectively.

Table S8. Conversion of interval categorical data on number of overnight trips to a continuous variable, related to Methods.

Question:
How many birdwatching trips requiring overnight accommodation in the Murray-Darling Basin would you normally take in a year (excluding zoos)?

**Categories presented in the survey**	**Number of overnight trips per year**
Five or more trips per year	5
Four trips a year	4
Three trips a year	3
Two trips a year	2
One trip per year	1
No overnight trips	0

Table S9. Conversion of interval categorical data on the number of day trips to a continuous variable, related to Methods.

Question:
How many birdwatching day trips (not requiring overnight accommodation) in the Murray-Darling Basin would you normally take in a year (excluding zoos)?

**Categories presented in the survey**	**Number of day trips per year**
More than two trips a week	144[#]
One or two trips a fortnight	39[##]
One or two trips a month	18[###]
Every couple of months	6
Three or four trips a year	3.5
About two trips a year	2
About one trip per year	1
Only very occasionally	0.5[§]

[#] Assume 3 trips per week for 48 weeks over one year (excluding Christmas and Easter holiday periods).
[##] Assume an average of 1.5 trips per fortnight over 26 fortnights i.e., 39 trips per year.
[###] Assume an average of 1.5 trips per month over 12 months i.e., 18 trips per year.
[§] Assume one trip every two years.

Per-trip expenditures

As shown in Figure S18, our survey includes questions on accommodation cost ($/person/night) for a typical overnight trip, expenditure on food and drink ($/person/day), fuel cost ($/day) and ‘other expenses’ ($/person/day). In Figure 5, we report the median value for each expenditure category as reported by survey respondents, categorised into eBird users who travel alone, as a pair or in a group of 2-5; and similarly for non-eBird users who travel alone, as a pair or in a group of 2–5. We did not include expenditure data from respondents who said they normally travelled in groups of 6–10, 11–20 or over 20 people because we were not convinced that expenditure data from a single respondent were necessarily representative of expenditures by all group members in these larger groups.

We recognise that eBird users who post frequently will be over-represented in BuD counts, whereas those who post only rarely will be under-represented. The box and whisker plots in Figure S20 show the distribution of expenditures across the four classes of eBird engagement intensity. Only modest differences in expenditure are present across the span of eBird engagement intensity for all expenditure categories. In our expenditure calculations, we therefore use the median expenditures of all eBird users, without sub-categorisation.

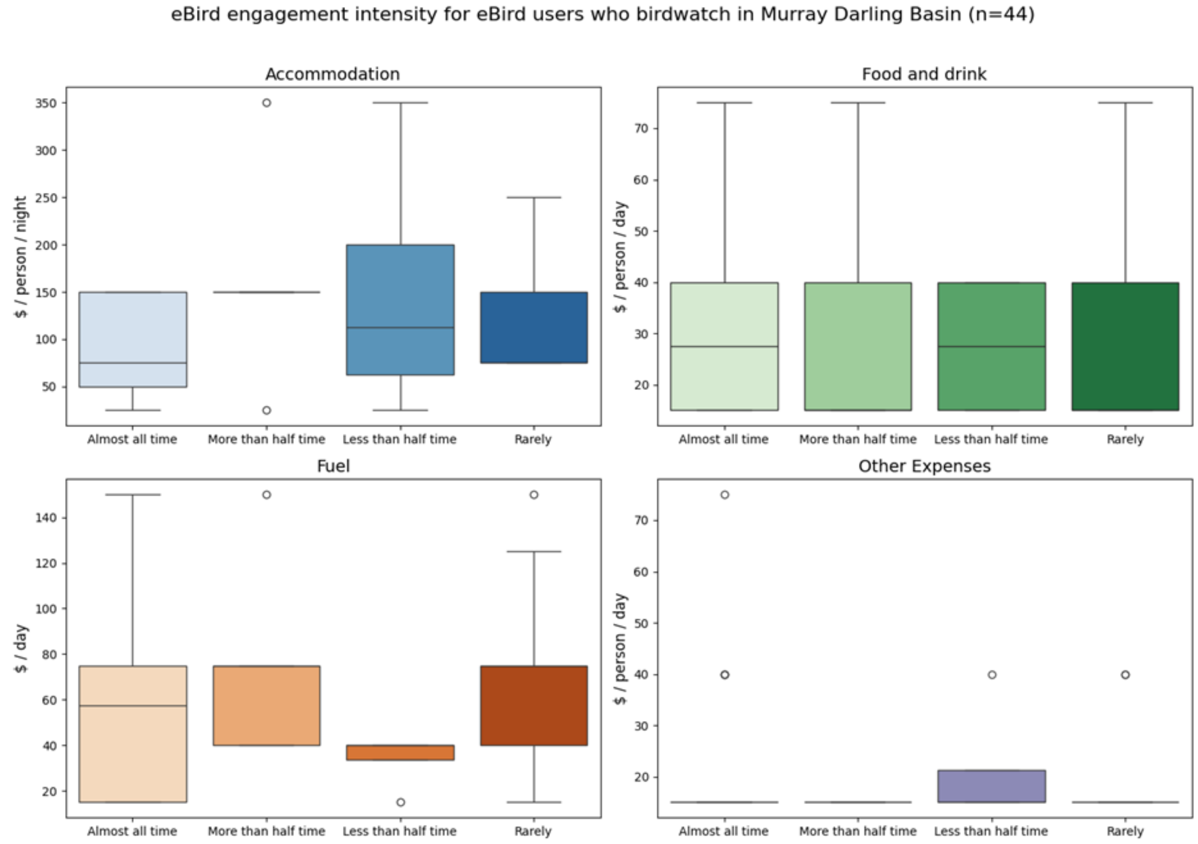

Figure S20. Trip expenditures categorised by levels of eBird engagement intensity for eBird users who birdwatch in the Murray-Darling Basin, related to Figure 5.

Box limits are the 25th and 75th percentiles with a horizontal line within the box indicating the median. The difference between the 25th and 75th percentiles is termed the interquartile range (IQR). Small circles are data that fall outside ± (1.5 x IQR) whiskers, or the extremity of the data (whichever comes first).

Annual expenditures by eBirders and non-eBirders by travelling group category

We use information from Figure 5 to calculate individual expenditures on birdwatching day trips per year and birdwatching overnight trips per year for eBird users and non-eBird users who go birdwatching in the Murray-Darling Basin, further categorised by travelling group size. Our expenditure calculations on day trips per year and overnight trips per year for eBirders and non-eBirders who go birdwatching in the Murray-Darling Basin utilise information on the median reported numbers of days trips and overnight trips per year for each travelling group size, together with median reported expenditures on food and drink, fuel, accommodation and ‘other’, (again categorised by eBird usage and travelling group size).

For all group sizes, one overnight trip is assumed to be a two-day trip with one overnight. For the 2-5 group size, four people are assumed to travel together in one vehicle with overnight accommodation as twin shares.

The resulting annual expenditures by category are:

- For eBirders (Figure 5) travelling:
  - alone: day trips $1710/year, overnight trips $680/year
  - as a pair: day trips $708/year, overnight trips $1008/year
  - as a group of 2-5: $347/year, $2982/year

- For non-eBirders (Figure 5) travelling:
  - alone: day trips $1710/year, overnight trips $265/year
  - as a pair: day trips $150/year, overnight trips $900/year

	eBird users: Total 37		
	Travel alone (27 respondents)	Travel as a pair (6 respondents)	Travel in 2-5 group (4 respondents)
No. of day trips per year	18	6	1
No. of overnight trips per year	2	3	3
Total no. trips per year	20	9	4
Annual activity proportions: day trips	**0.657**=27/37 x 18/20	**0.108**= 6/37 x 6/9	**0.027**= 4/37 x 1/4
Annual activity proportions: overnight trips	**0.073**=27/37 x 2/20	**0.054**= 6/37 x 3/9	**0.081**= 4/37 x 3/4

	Not eBird users: Total 12		
	Travel alone (7 respondents)	Travel as a pair (5 respondents)	Travel in 2-5 group (0 respondents)
No. of day trips per year	18	1	-
No. of overnight trips per year	2	2	-
Total no. trips per year	20	3	-
Annual activity proportions: day trips	**0.525**=7/12 x 18/20	**0.139**= 5/12 x 1/3	-
Annual activity proportions: overnight trips	**0.058**=7/12 x 2/20	**0.278**= 5/12 x 2/3	-

Figure S21. Annual activity proportions of day and overnight trips by each traveller grouping category for eBirders and non-eBirders from our survey sample, related to Figure 5 and Methods.

Birdwatching expenditures at hotspot sites

Figure S22 shows how probabilities derived from responses to survey questions on eBird usage and whether or not a respondent goes birdwatching in the Murray-Darling Basin can be combined to estimate the number of birdwatching visits to a site by eBird users *and* non-eBird users combined, when the number of eBirder visit days (BuDs) at that site is known. Following the logic in Figure S22, on average across survey respondents there is a 69.1% probability that someone who birdwatches in the Murray-Darling Basin is also an eBird user, and a 26.5% probability that someone who birdwatches in the Murray-Darling Basin is not an eBird user. This produces an extrapolation factor of 0.383 (= 26.5/69.1) from eBirder visit days (BuDs) to expected non-eBirder visit days. Thus, if the eBird annual BuD count at a site is 100, an additional 38.3 non-eBirder visits would also be expected at that site during the same year (100 x 26.5/69.1 = 38.3).

Table S9 reports results from extrapolating observed BuD counts to total expected birdwatching visitation, with accompanying expenditures, at the six hotspot sites where a direct or indirect association was identified between wet surface area and birdwatcher visitation. Annual BuD counts reported at those sites during 2018 (the most recent complete year in our BuD count dataset) are used as the basis for these calculations. Table S9 also reports the changes in birdwatching expenditure that would be expected to follow from a 15% increase in wet surface area at these sites.

The extrapolation factor of 0.383 between BuD count and the expected number of non-eBirder visit days is highly influential over the predicted expenditures in Table S9. This extrapolation factor was derived from our survey sample of Birdlife Australia's local group members.

Our survey finds that 72.3% of survey respondents used the eBird app for logging their species sighting lists. This level of usage appears rather high for one bird species logging app, particularly when several alternative apps with similar species logging capabilities are available (e.g., Birdata: https://birdata.birdlife.org.au , birda: https://birda.org , Merlin: https://merlin.allaboutbirds.org , bird journal: https://www.birdjournal.com). A high level of reported eBird usage acts to reduce the extrapolation factor (i.e., 1/eBird_usage) for estimating annual birdwatching-related expenditures and expenditure increases.

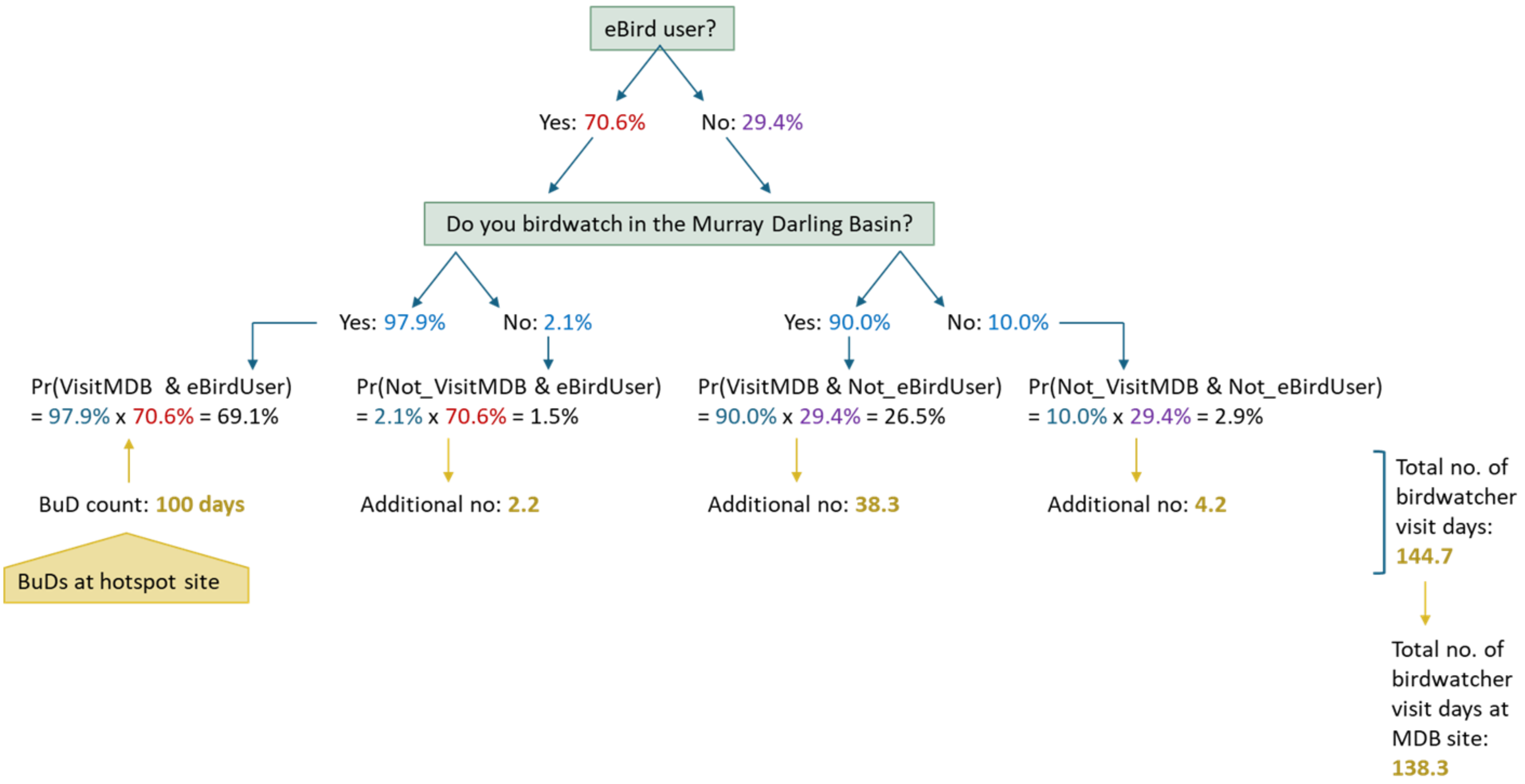

Figure S22. Calculation sequence in determining the expected number of non-eBirder visits at a hotspot site, knowing the annual BuD count at that site, related to Methods.

The probabilities are based on the responses received from the online survey from respondents (n = 68) who answered the questions 'Have you heard of the eBird website where you can post your bird sightings online (www.eBird.org) ? [no, yes but I don't use it, yes]' and 'Do you birdwatch on waterways, lakes, wetlands or other types of sites within the Murray-Darling Basin? [no, yes]'. The prediction following from an annual BuD count of 100 BuDs is provided for illustration only.

Table S10. Total annual birdwatching-related expenditures at hotspot sites extrapolated from monthly BuD counts at those sites during calendar year 2018 using an extrapolation factor of 0.383 from annual BuD count to non-eBirder visit days, related to Figure 6A-B and Methods.
This extrapolation factor was derived from responses to a survey conducted with members of Birdlife Australia local birdwatching groups.

Hotspot	Total annual BuD count 2018	Extrapolated non-eBirder visit days in 2018	Annual birdwatching expenditure from BuD counts in 2018 ($1000s)	Annual birdwatching expenditure from non-eBirder visit days in 2018 ($1000s)	Total annual extrapolated birdwatching expenditure in 2018 ($1000s)	Expected change in 2018 birdwatching expenditure following a 15% increase in wet area in each preceding month ($1000s)
Barmah Murray Valley National Parks	84	32	8.2	3.2	11.4	
Bowra Wildlife Sanctuary	398	153	39.1	15.2	54.2	11.9
Lake Cargelligo	110	42	10.8	4.2	15.0	4.7
Fivebough Swamp and Tuckerbill Wetland	69	26	6.8	2.6	9.4	ns
Gum Swamp	44	17	4.3	1.7	6.0	3.0
Hattah Kulkyne National Park	288	111	28.3	11.0	39.2	
Kerang Wetlands	237	91	23.3	9.0	32.3	3.3[#]
Macquarie Marshes Nature Reserve	31	12	3.0	1.2	4.2	2.3
Shepparton	329	126	32.3	12.5	44.8	-4.7
Wonga and West Albury Wetlands	136	52	13.3	5.2	18.5	

Totals may not sum exactly due to rounding. Grey cells in column 7 indicate that wet area in the preceding year did not feature in the best-fitting regression model for this hotspot. ‘ns’ indicates that wet area in the preceding year was not a significant driver of BuD count at this hotspot.
[#] At Kerang Wetlands the relationship between wet area and BuD count is mediated through an increase in species richness. The predicted increase in birdwatching expenditure at Kerang includes the effect of increased wet area two months prior on species richness one month prior, and the subsequent effect of increased species richness on increased BuD count in the current month.

Birdwatching expenditures from our survey compared with birdwatching expenditures from Tourism Research Australia's Domestic National Visitor Survey

Tourism Research Australia's (TRA's) Domestic National Visitor Survey included birdwatching as a category of outdoor recreation from 2019 onwards (Steven 2022[5]). Steven quotes TRA results for birdwatchers' average expenditure on a day trip ($89) and per day of an overnight trip ($181). Converting these to FY2024-25 AUD$ using the Reserve Bank of Australia's inflation calculator (https://www.rba.gov.au/calculator/financialYearDecimal.html) produces average day trip and per day of overnight trip expenditures of $109 and $223, respectively. These figures can be compared with average per day and per day of overnight trip expenditures calculated from our birdwatchers' online survey.

Average per person day trip expenditure and average per person per day of overnight trip expenditure can be calculated from survey responses knowing the per person per day expenditures incurred by eBirders and non-eBirders combined when travelling alone, as a pair, or as a group of two to five people (Figure S21). The resulting expenditures from our survey in FY2024-25 AUD$ are:

average per person day trip expenditure = $88.39 ± $3.53 (95% CI)

average per person per day of overnight trip expenditure = $207.78 ± $14.44 (95% CI)

Figure S23 compares day trip and per day of overnight trip expenditures from our survey with those from TRA's FY2018-19 Domestic National Visitor Survey (expressed in FY2024-25 AUD$). Figure S23 suggests that, whilst not identical, expenditure results from our small visitor survey are comparable with average expenditure results from the 516,000 birdwatching day trips and 331,000 birdwatching overnight trips taken by domestic visitors reported in TRA's Domestic National Visitor Survey (as quoted in Steven 2022).[S6]

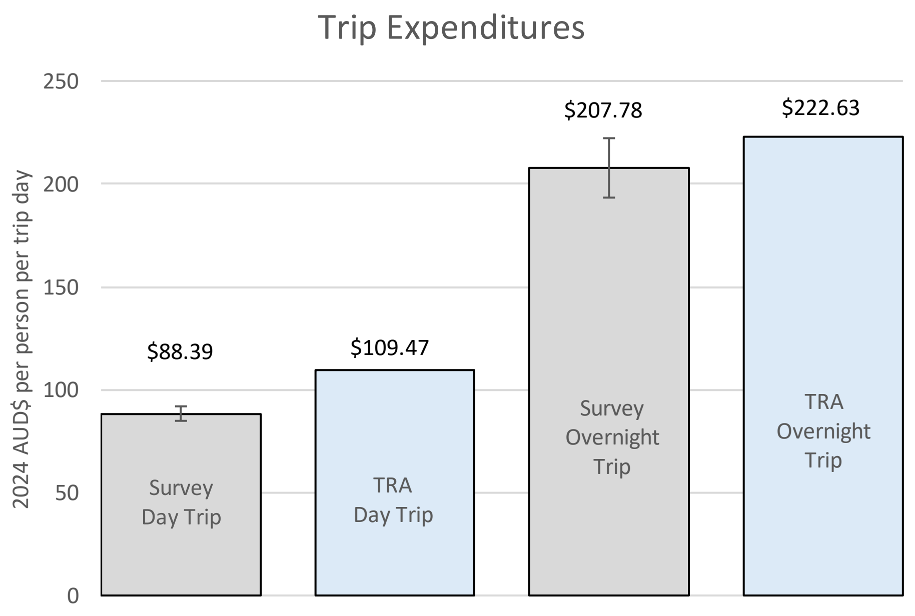

Figure S23: Comparison of mean day trip and mean per day of overnight trip expenditures from our survey of birdwatchers in local birdwatching groups with those from TRA's FY2018-19 Domestic National Visitor Survey (expressed in FY2024-25 AUD$). 95% confidence intervals around mean expenditure estimates from our survey are shown on the plot, related to Discussion.

References

S1. Dunn, B., Krause, C., Newey, V., Lymburner, L., Alger, M.J., Adams, C., Yuan, F., Ma, S., Barzinpour, A., Ayers, D., McKenna, C., Schenk, L., (2024) Digital Earth Australia Waterbodies Version 3. Commonwealth of Australia (Geoscience Australia). https://dx.doi.org/10.26186/148920

S2. Krause, C.E., Newey, V., Alger, M.J., and Lymburner, L. (2021). Mapping and Monitoring the Multi-Decadal Dynamics of Australia's Open Waterbodies using Landsat. Remote Sensing (Basel) *13*, 1437. https://doi.org/10.3390/rs13081437

S3. Garnett, S.T., Duursma, D.E., Ehmke, G., Guay, P.J., Stewart, A., Szabo, J.K., Weston, M.A., Bennett, S., Crowley, G.M., Drynan, D., et al. (2015). Biological, ecological, conservation and legal information for all species and subspecies of Australian bird. Sci Data *2*. https://doi.org/10.1038/sdata.2015.61.

S4. Smart, J. C. R. and Harte, J. (2024). Murray-Darling Basin Authority Water & Environment Research Program Theme 4: Research Question 12.2, Deliverable 12.2.6 Recreational and tourism value of healthy rivers: Extension A – Final Report. Australian Rivers Institute, Griffith University, Brisbane. https://www.mdba.gov.au/sites/default/files/publications/recreational-and-tourism-value-of-healthy-rivers-extension-a-final-report.pdf

S5. Sullivan, B.L., Wood, C.L., Iliff, M.J., Bonney, R.E., Fink, D., and Kelling, S. (2009). eBird: A citizen-based bird observation network in the biological sciences. Biol Conserv *142*, 2282–2292. https://doi.org/https://doi.org/10.1016/j.biocon.2009.05.006.

S6. Steven, R. (2022). Bird and Nature Tourism in Australia. KBAs in Danger Case Study Report. https://researchportal.murdoch.edu.au/esploro/outputs/report/Bird-and-Nature-Tourism-in-Australia/991005542577807891.
